# Urinary stress hormones and their metabolites as predictive biomarkers for CKD with diabetes

**DOI:** 10.1186/s12882-025-04717-9

**Published:** 2025-12-24

**Authors:** Yujie Jin, Song Dong, Yan Ma, Chunchen Ni, Yan Yao, Shujuan Shang, Mengru Wang, Jiahao Xu, Fan Liu, MengMeng Qian, Shiqiang Liu, Dong Li, Lizhuo Wang, Liuming Yu, Jialin Gao

**Affiliations:** 1 Department of Endocrinology and Genetic Metabolism, The First Affiliated Hospital of Wannan Medical College (Yijishan Hospital of Wannan Medical College), Wuhu, Anhui China; 2 Institute of Endocrine and Metabolic Diseases, The First Affiliated Hospital of Wannan Medical College (Yijishan Hospital of Wannan Medical College), Wuhu, Anhui China; 3 Anhui Province Key Laboratory of Basic d Research an Transformation of Age-Related Diseases, Wannan Medical College, Wuhu, Anhui China; 4 Department of Biochemistry and Molecular Biology, Wannan Medical College, Wuhu, Anhui China; 5 Suzhou Boyuan Medical Technology Co., LTD, Suzhou, Jiangsu China

**Keywords:** Type 2 diabetes mellitus, CKD with diabetes, Urinary stress hormones, Adrenal hormones, Biomarkers

## Abstract

**Aims:**

This study aimed to characterize urinary stress hormones and their metabolites in patients with chronic kidney disease associated with diabetes (CKD with diabetes) and to evaluate their associations with renal function, providing insights into stress-related mechanisms and potential biomarker utility.

**Materials and methods:**

We enrolled 735 participants, including 449 with type 2 diabetes mellitus(T2D)and 286 with CKD with diabetes. Urinary concentrations of norepinephrine, cortisol, aldosterone, and 17-ketosteroids were analyzed. Statistical methods included correlation and regression analyses, receiver operating characteristic (ROC) curves, and orthogonal partial least squares discriminant analysis (OPLS-DA) to evaluate diagnostic value.

**Results:**

Urinary norepinephrine, cortisol, and 17-ketosteroids levels were significantly lower in CKD patients with diabetes than in those with diabetes alone. Norepinephrine was inversely correlated with albumin-to-creatinine ratio, urinary microalbumin, blood urea nitrogen, and serum creatinine, and positively with estimated glomerular filtration rate. Similar trends were observed for cortisol and 17-ketosteroids. Aldosterone was also negatively correlated with urinary microalbumin and creatinine. OPLS-DA showed distinct metabolic profiles between CKD with diabetes and diabetes, suggesting metabolic heterogeneity. Multivariate logistic regression identified norepinephrine as an independent protective factor, while diastolic blood pressure, urinary glucose, and homovanillic acid were risk factors. A composite model integrating norepinephrine, 17-ketosteroids, and homovanillic acid demonstrated high diagnostic performance, with the norepinephrine-based model achieving an AUC of 0.831 in validation.

**Conclusion:**

Urinary adrenal hormones and their metabolites provide valuable insights into stress-related mechanisms in CKD with diabetes and may hold potential as complementary noninvasive biomarkers for disease assessment.

**Clinical trial number:**

Not applicable.

**Supplementary Information:**

The online version contains supplementary material available at 10.1186/s12882-025-04717-9.

## Introduction

Chronic kidney disease associated with diabetes (CKD with diabetes) is one of the most prevalent and serious complications of diabetes mellitus (DM) and has become a leading cause of chronic kidney disease and end-stage renal disease worldwide [1, 2]. The pathogenesis and progression of CKD with diabetes are multifactorial, involving sustained hyperglycemia, insulin resistance, dyslipidemia, oxidative stress, and chronic inflammation [3, 4]. Although current therapeutic strategies, such as glycemic control and antihypertensive treatment, can delay CKD with diabetes progression, significant clinical challenges remain.

The diagnosis of CKD with diabetes currently relies on conventional biomarkers, such as the urinary albumin-to-creatinine ratio (UACR) and estimated glomerular filtration rate (eGFR). Although these indicators remain fundamental for clinical evaluation, they do not capture the full spectrum of metabolic and molecular alterations that accompany CKD with diabetes [5, 6]. Therefore, there remains a need to identify biomarkers that more accurately reflect the underlying pathophysiological processes and complement existing clinical indicators. Among emerging candidates, urinary metabolites—particularly those related to stress hormone pathways—offer distinct advantages in capturing systemic metabolic dysregulation and neuroendocrine stress responses associated with CKD with diabetes. Elucidating these metabolic alterations may deepen our understanding of disease mechanisms and facilitate more refined evaluation and management of CKD with diabetes.

Adrenal hormones play a critical role in mediating the body’s stress responses [7]and are also involved in the pathophysiology of DM [5]. Increasing evidence suggests that diabetic patients often exhibit chronic activation of the stress axis, and dysregulated adrenal hormone levels may contribute to the onset and progression of CKD with diabetes. In CKD with diabetes, aberrant adrenal hormone secretion may promote renal inflammation, fibrosis, and oxidative injury, thereby exacerbating tubular damage and accelerating kidney dysfunction [6]. Urinary adrenal hormones and their metabolites, as sensitive markers of systemic stress, may be used to assess renal involvement in stress responses and provide valuable insights into the pathogenesis of CKD with diabetes.

Accordingly, the present study aimed to investigate alterations in urinary adrenal hormone profiles in patients with CKD with diabetes and to explore their associations with renal function and metabolic abnormalities, providing insight into stress-related endocrine and metabolic mechanisms underlying disease progression.

## Methods

### Study population

A total of 735 eligible participants were enrolled in this study, including 449 patients diagnosed with type 2 DM (DM group) and 286 patients with CKD with diabetes (CKD with diabetes group). Participant recruitment was conducted from December 1, 2022, to December 31, 2024.The study protocol was approved by the Ethics Committee of Wannan Medical College (Approval No. 2024-61).

### Inclusion criteria

Type 2 DM was diagnosed according to the 2021 American Diabetes Association Diagnostic Criteria [8], while CKD with diabetes was defined following the 2020 Kidney Disease: Improving Global Outcomes Clinical Practice Guideline [9].

### Exclusion criteria

Participants were excluded if they met any of the following criteria: (1) Presence of mitochondrial gene mutations or other types of diabetes or kidney disease; (2) Recent occurrence of acute diabetic complications, trauma, inflammation, or systemic infections; (3) Diagnosis of concomitant hematological disorders, rheumatologic diseases, or autoimmune conditions; (4) Pregnancy or lactation; (5) Ongoing participation in interventional clinical trials or unwillingness to cooperate; (6and clopidogrel was comparable) Ongoing renal replacement therapy; (7) History of diabetic ketoacidosis or hyperosmolar hyperglycemic syndrome.

### Sample collection and laboratory testing

**Fig. 1:**
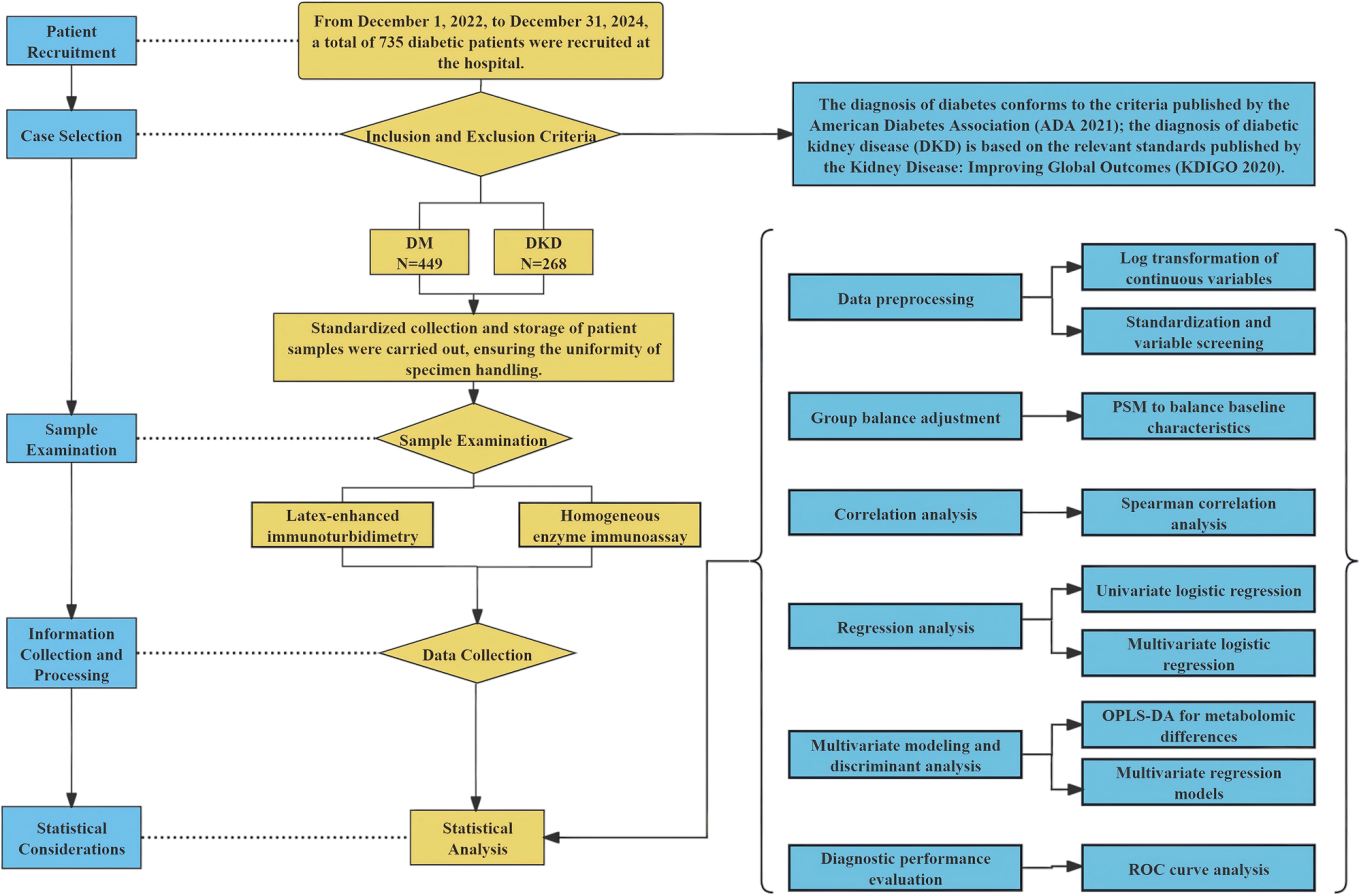
Overall workflow of patient recruitment, sample examination, and statistical analyses. The flowchart illustrates the screening and enrollment of participants, subsequent collection and processing of clinical and laboratory data, and the statistical analyses performed in the study

First-morning midstream urine samples were immediately pre-processed by centrifugation at 2000 x g for 10 min, followed by aliquoting into 10 mL tubes and storage at -80℃. A minimum volume of 200 µL was required for each assay. All specimens were transported on dry ice within 24 h to Boyuan Laboratory (Suzhou, China) for centralized testing. Urinary adrenal hormones and metabolites were quantified using commercial reagent kits (Suzhou Boyuan Medical Technology Co., Ltd., China) on a fully automated biochemical analyzer (Hitachi 7180, Hitachi, Japan). Specifically, vanillylmandelic acid (VMA), 17-ketosteroids (17-KS), 17-hydroxycorticosteroids (17-OHCS), and cortisol (COR) were measured by homogeneous enzyme immunoassay using two-component reagents (R1 and R2). Homovanillic acid (HVA) and aldosterone (ALD) were determined by the same method, whereas norepinephrine (NE) and epinephrine (E) were assayed by latex-enhanced immunoturbidimetry. Urinary creatinine was measured by homogeneous enzyme immunoassay, and urinary albumin was measured by immunoturbidimetry. The albumin-to-creatinine ratio (ACR) was subsequently calculated, and hormone concentrations were normalized to urinary creatinine. Urinary hormone concentrations were normalized to creatinine to correct for dilution effects, as urine osmolarity data were unavailable for all samples.Detailed reagent specifications are provided in Supplementary Table S1. The overall workflow of participant recruitment, sample collection, laboratory testing, and statistical analysis is summarized in Fig. 1.

### Statistical analysis

Continuous variables were log-transformed to approximate normalized distributions. Associations between urinary metabolites and renal function indicators were evaluated using Spearman correlation analysis. Prior to univariate logistic regression, all variables were standardized and screened. Model fit was assessed using the Hosmer-Lemeshow test, and odds ratios (ORs) with 95% confidence intervals (CIs) were reported. Key metabolites were included in multivariate logistic regression models to adjust for potential confounders and determine independent associations. Propensity score matching was applied to balance baseline characteristics between the DM and CKD with diabetes groups. Orthogonal partial least squares discriminant analysis (OPLS-DA) was conducted to identify metabolic differences between groups. Multivariate regression models were developed, and receiver operating characteristic (ROC) curve analysis was used to evaluate diagnostic performance (training set: 82%; validation set: 18%). An area under the curve (AUC) above 0.7 was considered good diagnostic efficacy. Comparisons of continuous variables were performed using the Mann-Whitney U test or Student’s *t*-test, depending on data distribution. Categorical variables were analyzed using the Chi-square test or Fisher’s exact test. Results are presented as mean ± standard deviation or median (interquartile range), as appropriate. A two-sided *P*-value of less than 0.05 was considered statistically significant. All statistical analyses were performed using SPSS version 26.0 and GraphPad Prism version 9.0.

## Results

### General and clinical characteristics of the study population

A total of 735 participants were included in the analysis, with 449 in the DM group and 286 in the CKD with diabetes group. There were no significant differences in age or gender distribution between the two groups. Compared with the diabetes group, patients with CKD with diabetes had a longer disease duration, higher body mass index, and elevated blood pressure, with a markedly higher prevalence of hypertension.

In laboratory findings, CKD with diabetes patients exhibited impaired renal function, reflected by increased serum creatinine, urea nitrogen, uric acid, and cystatin C levels, along with decreased serum albumin. Inflammatory changes were also suggested by higher white blood cell and platelet counts. Glycemic control indicators, including HbA1c and fasting glucose, did not differ significantly between the groups.Regarding concomitant medications, the use of ACEI/ARB, dapagliflozin, insulin, statins, and aspirin was more frequent in the CKD with diabetes group, whereas the use of metformin and clopidogrel was comparable between groups. Comprehensive clinical and laboratory characteristics are summarized in Table 1, and the distribution of past medical history is provided in Supplementary Table S2.

**Table 1:** Comparison of baseline characteristics between patients with diabetes mellitus and CKD with diabetes

General characteristics		DM	CKD with diabetes	z/χ²	*P*
Age					
	< 40	59(13.5%)	37(13.3%)	0.08	0.959
	≥ 40, < 60	192(44%)	120(43.2%)		
	≥ 60	185(42.4%)	121(43.5%)		
Sex				0.03	0.864
	Male	251(57.8%)	162(58.5%)		
	Female	183(42.2%)	115(41.5%)		
Disease duration				12.77	0.002*
	≤ 5	191(53.7%)	84(38.5%)		
	6–10	92(25.8%)	70(32.1%)		
	> 10	73(20.5%)	64(29.4%)		
BMI,				15.79	0.001*
	< 18.5	13(5.2%)	6(3.6%)		
	18.5 ≤ BMI < 23.9	140(55.6%)	66(39.1%)		
	24 ≤ BMI < 27.9	77(30.6%)	66(39.1%)		
	BMI ≥ 28	22(8.7%)	31(18.3%)		
SBP		128(119,141)	142(128.75,153.25)	-6.26	0*
DBP		79(72,86)	82(75,91)	-3.23	0.001*
Glycometabolism index	HbA_1_c	7.60(6.60, 10.0)	7.60(6.60, 9.30)	-0.73	0.466
	FBG	7.55(6.41, 9.32)	8.07(6.38, 10.31)	-1.4	0.162
Renal function	BUN	5.34(4.31,6.74)	5.89(4.88,6.74)	-3.09	0.002*
	Cr	71.8(56.30, 80.30)	76.85(66.73, 96.53)	-5.64	0*
	UA	283.1(224.40,357.7)	311(259.55,391.45)	-3.57	0*
	Cystatin-C	0.78(0.66, 0.95)	0.93(0.73, 1.28)	-5.39	0*
Liver function	Albumin	44.60(41.50, 46.90)	43.20(40.03, 46.28)	-2.81	0.005*
	DBil	3.81(2.84, 4.87)	3.39(2.50, 4.74)	-3.95	0*
	ALT	18.00(13.00, 26.00)	18.00(14.0, 33.50)	-0.92	0.356
	AST	19.00(15.00, 22.00)	18.50(14.0, 26.00)	-0.16	0.871
Blood fat	TC	4.62(3.85,5.15)	4.64(3.92, 5.51)	-1.9	0.058
	TG	1.43(0.85, 2.12)	1.60(1.04, 2.60)	-4.95	0*
	HDL	1.24(1.03,1.50)	1.23(1.06, 1.47)	-0.52	0.607
	LDL	2.57 ± 0.81	2.77 ± 1.02	-1.87	0.062
Routine blood test	WBC	6.00(5.10,7.20)	6.75(5.70,8.48)	-4.58	0*
	RBC	4.53 ± 0.56	4.47 ± 0.75	-0.63	0.528
	Hb	137.03 ± 17.12	134.33 ± 21.61	0.17	0.867
	PLT	172.0(141.00, 210.00)	203(141,210)	-3.3	0.001*

Note: (1) * means *P* < 0.05, with statistically significant difference. (2) the data is expressed in median (quartile), and the specific form depends on the distribution characteristics of the data

### Comparison of urinary stress hormones between CKD with diabetes and DM groups and OPLS-DA model analysis

Urinary Stress Hormones levels were compared between the DM and CKD with diabetes groups to assess alterations in stress hormone profiles. Among catecholamines, urinary NE levels were significantly lower in the CKD with diabetes group compared to the DM group. However, no significant differences were observed in E, HVA, or VMA between the two groups. Regarding glucocorticoids, the CKD with diabetes group exhibited significantly lower urinary levels of 17-KS, 17-OHCS, and COR compared to the DM group. For ALD levels were also significantly reduced in the CKD with diabetes group. A summary of these results is shown in Table 2.

**Table 2:** Comparative analysis of urinary stress hormone between CKD with diabetes **and DM group**

Urinary stress hormones		DM	CKD with diabetes	Z	*P*
Catecholamine					
	Norepinephrine	208.87(171.08, 253.43)	173.46(130.94, 234.64)	-5.46	0*
	Adrenaline	63.22(51.08, 83.52)	63.45(51.5, 84.47)	-0.27	0.791
	Homovanillic acid	4.86(3.57, 6.47)	4.85(3.70, 7.12)	-1.52	0.128
	Vanillic mandelic acid	6.04(4.85, 7.73)	5.63(4.26, 7.80)	-1.82	0.07
Glucocorticoid					
	17-ketosteroids	6.45(4.68, 8.84)	5.58(4.04, 7.41)	-3.44	0.001*
	17-hydroxysteroid	5.18(4.00, 6.71)	5.38(4.19, 7.39)	-2.01	0.044*
	Cortisol	307.98(215.55, 414.37)	265.49(178.09, 374.94)	-2.62	0.009*
Mineralocorticoid					
	Aldosterone	8.22(6.23, 10.66)	7.35(5.83, 9.97)	-2.1	0.036*
Routine indicators of diabetes					
	β - hydroxybutyric acid	16.97(12.19, 26.27)	17.23(12.17, 27.13)	-0.65	0.513
	Urine glucose	8.82(0.96, 161.26)	60.05(3.2, 153.63)	-2.82	0.005*
	NAG	15.72(7.79, 24.5)	13.0(6.38, 27.0)	-1.14	0.253
Urine protein					
	Total protein	7.0(4.60, 10.05)	28.1(13.625, 62.85)	-18.15	0*
	Retinol binding protein	3.83(0.46, 7.79)	5.54(0.38, 18.85)	-3.97	0*
	Immunoglobulin G	6.36(3.89, 10.1)	19.41(9.59, 37.12)	-14.59	0*
	Kappa light chain	2.81(1.36, 6.1)	10.68(4.57, 22.51)	-12.99	0*
	Lambda light chain	0.93(0.38, 1.91)	6.77(2.99, 14.34)	-17.83	0*
	Transferrin	7.92(0.97, 18.03)	15.62(6.68, 25.24)	-7.92	0*
	α 1-microglobulin	0.2(0.05, 3.37)	3.46(0.14, 13.54)	-7.61	0*
	β 2-microglobulin	0.19(0.10, 0.39)	0.27(0.09, 0.89)	-3.11	0.002*

Note: * indicates *P* < 0.05, with statistically significant difference

**Fig. 2:**
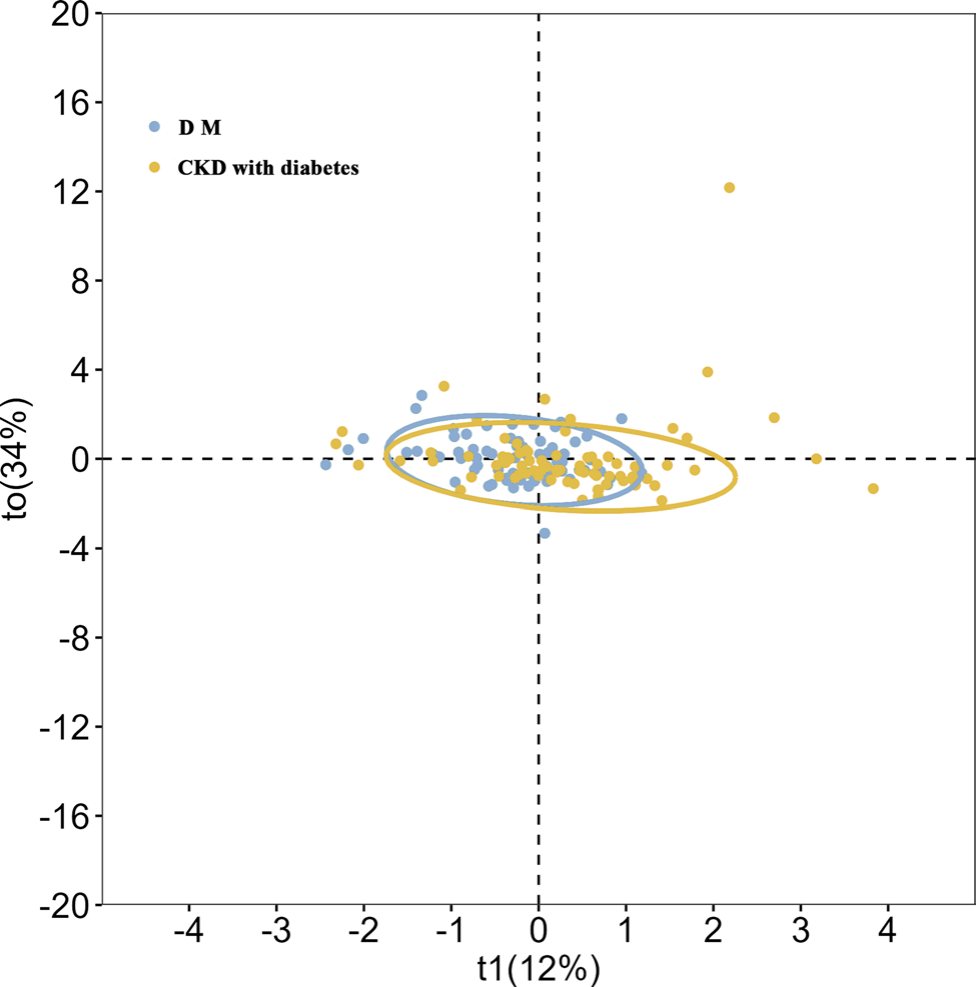
OPLS-DA score map of metabolic differences between CKD with diabetes group and DM group. OPLS–DA score plot differentiating diabetes mellitus (DM, blue) and diabetic kidney disease (CKD with diabetes, yellow) groups based on urinary metabolomic profiles. Orthogonal partial least squares discriminant analysis (OPLS–DA) was performed to explore metabolic differences. The plot shows sample distributions along the first predictive component (t1, 12% variance explained) and the first orthogonal component (t0, 34% variance explained). Each point represents an individual sample, and 95% confidence ellipses indicate group clustering. A separation trend between DM and CKD with diabetes groups was observed, although partial overlap persisted, reflecting shared metabolic features and intergroup heterogeneity

To further explore urinary metabolic differences between the DM and CKD with diabetes groups and assess their predictive potential, OPLS–DA was performed. The OPLS–DA score plot suggested a separation trend between the DM and CKD with diabetes groups; however, overlap was present, indicating that the metabolic distinction between groups was not absolute **(**Fig. 2**).** This overlap likely reflects shared metabolic features and underlying heterogeneity. The CKD with diabetes group exhibited relatively tighter clustering of data points, suggesting more homogeneous metabolic characteristics, whereas the DM group showed a more dispersed distribution, indicating greater intragroup variability. Confidence ellipse analysis supported these findings, showing larger variability within the DM group and higher consistency within the CKD with diabetes group.

### Differences in urinary stress hormones between DM and CKD with diabetes groups stratified by UACR and other variables

**Fig. 3:**
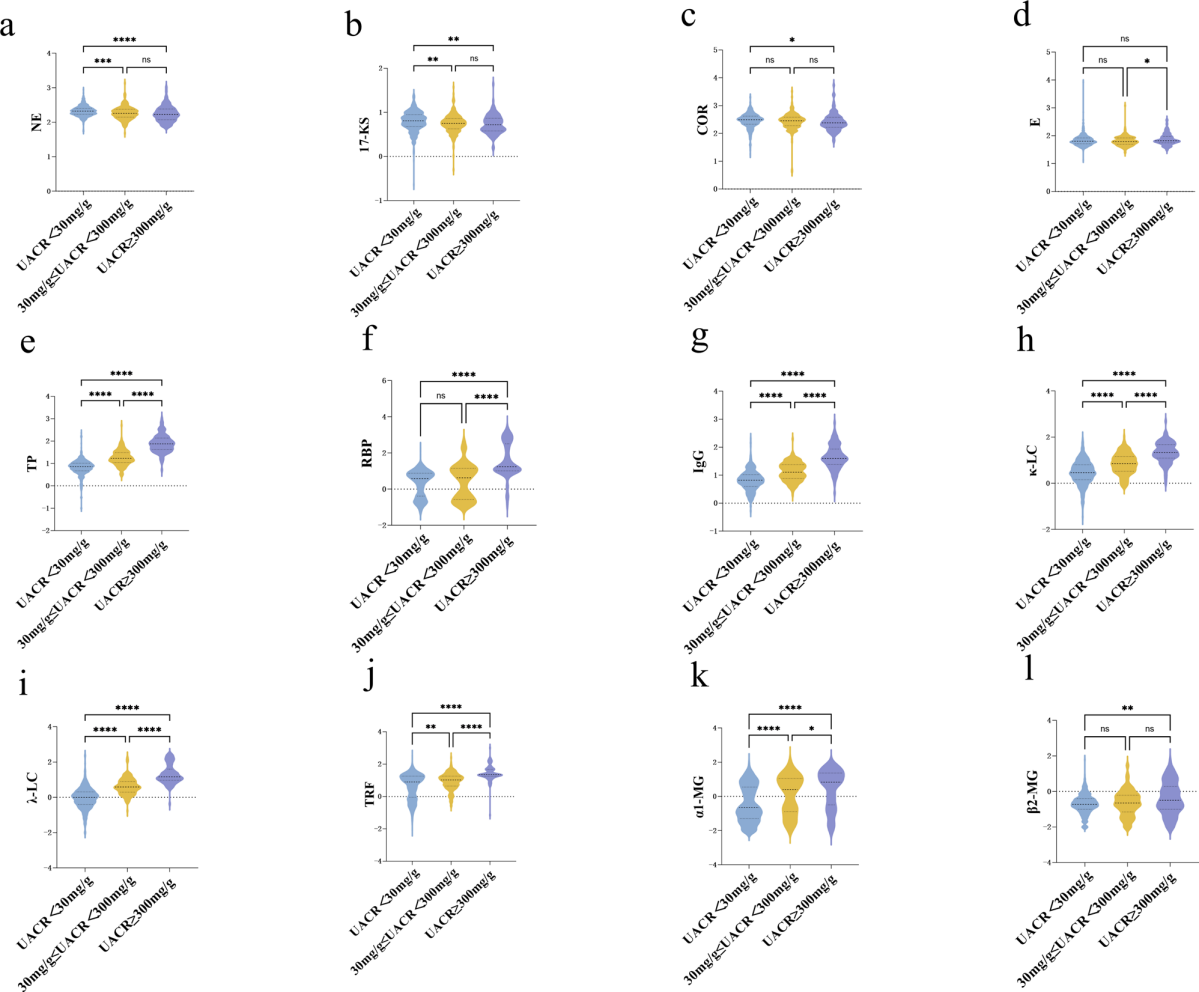
Difference of urinary stress hormone under different ACR stratification. Differences in urinary stress hormone levels across ACR stratifications.Violin plots display the log-transformed distributions of twelve urinary adrenal hormone metabolites across three albumin-to-creatinine ratio (UACR) groups. Statistical differences were evaluated using Dunn’s multiple comparisons test. Asterisks indicate statistical significance: *P* < 0.05 (*), *P* < 0.01 (**), *P* < 0.001 (***), *P* < 0.0001 (****); “ns” denotes non-significant differences (*P* > 0.05).Panel descriptions: (**a**) NE, (**b**) 17-KS, (**c**) COR, (**d**) E, (**e**) TP, (**f**) RBP, (**g**) IgG, (**h**) κ-LC, (**i**) λ-LC, (**j**) TRF, (**k**) α1-MG, (**l**) β2-MG.The width of each violin represents kernel density estimation of the data distribution. Boxes within the violins denote the interquartile range with the median line shown

Stratified analysis based on UACR revealed distinct trends in urinary adrenal hormone levels. NE demonstrated a significant, stepwise decline with increasing UACR levels. The highest concentration of NE was observed in the UACR < 30 mg/g subgroup, while the lowest was found in the UACR ≥ 300 mg/g subgroup. In contrast, E, VMA, and HVA did not exhibit significant intergroup differences across UACR strata. Regarding glucocorticoids, both 17-KS and COR declined significantly with increasing UACR, whereas 17-OHCS did not differ significantly. ALD also showed no significant differences between UACR groups. Detailed results are presented in Fig. 3; Table 3.

**Table 3:** Comparative analysis of urinary stress hormones in different UACR groups

Urinary stress hormones	UACR<30 mg/g	30 mg/g ≤ UACR<300 mg/g	UACR ≥ 300 mg/g	H	*P*
Norepinephrine	208.96 (171.26, 253.22)	180.02 (139.64, 237.34)	164.41 (118.79, 240.40)	27.86	0*
Adrenaline	63.22 (51.18, 83.54)	61.33 (49.29, 83.54)	66.00 (54.63, 92.22)	6.11	0.047*
Homovanillic acid	4.86(3.62, 6.50)	4.78 (3.71, 7.42)	5.13 (3.69, 7.26)	2.26	0.323
Vanillic mandelic acid	6.04(4.86,7.60)	5.75(4.44,7.80)	5.72(4.28,7.97)	1.6	0.449
17-ketosteroids	6.44 (4.73, 8.82)	5.55 (4.18, 7.18)	5.08 (3.83, 7.20)	15.48	0*
17-hydroxysteroid	5.20(4.03,6,75)	5.44(4.28,7.39)	5.23(3.86,7.58)	3.67	0.159
Cortisol	307.89 (216.14, 414.53)	282.43 (187.70, 382.21)	236.31 (166.91, 374.23)	9.12	0.010*
Aldosterone	8.22 (6.19, 10.68)	7.76 (5.81, 9.85)	7.05 (5.67, 9.99)	5.07	0.079
β - hydroxybutyric acid	16.66 (12.32, 26.29)	16.02(11.54, 25.02)	19.13 (13.26, 30.05)	5.49	0.064
Urine glucose	10.67 (0.98, 163.20)	47.86(1.54, 156.55)	66.08(8.01, 150.18)	6.66	0.036*
NAG	15.84(7.54, 24.50)	12.00 (6.02, 27.25)	14.41 (7.25, 27.50)	1.66	0.435
Total protein	7.10 (4.70, 10.00)	16.95 (10.88, 30.85)	76.10 (42.10, 142.55)	351.35	0*
Retinol binding protein	3.83 (0.41, 7.60)	4.13 (0.26, 13.94)	7.74 (0.80, 135.38)	24.23	0*
Immunoglobulin G	6.44(3.91, 10.10)	13.01 (7.53, 23.91)	39.56 (23.70, 87.26)	245.1	0*
Kappa light chain	2.84(1.36, 6.13)	7.11(3.34, 14.53)	21.98 (12.33, 48.31)	198.12	0*
Lambda light chain	0.93 (0.38, 1.92)	3.80 (1.95, 8.02)	14.93 (9.48, 42.55)	333.97	0*
Transferrin	7.86 (0.87, 17.99)	10.51 (4.42, 18.22)	23.08(18.34, 48.13)	104.72	0*
α 1-microglobulin	0.21(0.05, 3.49)	2.20 (0.10, 11.13)	6.42 (0.30, 22.98)	62.9	0*
β 2-microglobulin	0.19(0.09, 0.39)	0.22(0.07, 0.61)	0.35 (0.09, 1.94)	11.73	0.003*

Note: * indicates *P* < 0.05, with statistically significant difference

Gender-based analysis revealed consistently higher levels of NE, epinephrine, VMA, HVA, COR, and ALD in females compared to males, whereas males exhibited elevated levels of 17-KS and 17-OHCS. Age-related analysis showed that NE, 17-KS, and 17-OHCS levels significantly decreased with advancing age. Conversely, the levels of E, VMA, HVA, and kappa light chain progressively increased (Table S3). NE levels peaked in individuals under 40 years, declined moderately in those aged 40–59 years, and reached the lowest levels in the ≥ 60-year group. In contrast, epinephrine, VMA, and HVA reached their highest concentrations in the ≥ 60-year stratum. No significant age-dependent variations were observed in COR or ALD levels. Stratification by disease duration revealed a significant intergroup difference in urinary glucose (UGLU) levels, while other urinary metabolites showed no significant variations across different disease duration subgroups (Table S4). When stratified by disease duration and BMI, no further significant or clinically meaningful differences were observed. Detailed results are presented in Supplementary Tables S5-S6.

### Spearman correlation analysis: associations between urinary stress hormones and renal function

**Fig. 4:**
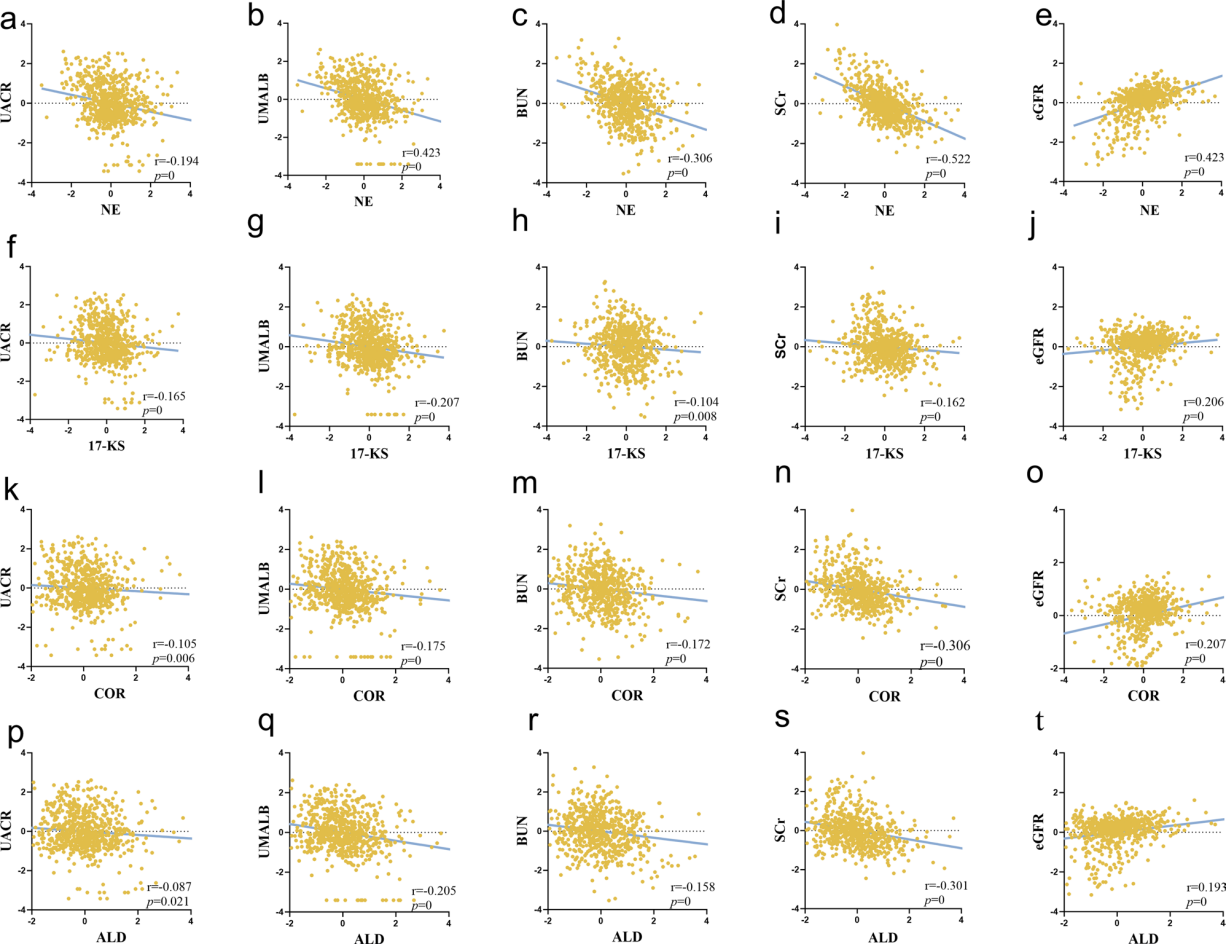
Assessment of the correlation between urinary stress hormone and renal function. Scatter plots illustrate correlations of NE with UACR, UMALB, BUN, sCr, and eGFR (**a–e**); 17-KS with UACR, UMALB, BUN, sCr, and eGFR (f–j); COR with the same renal indicators (**k–o**); and ALD with UMALB and sCr (p–t). NE and 17-KS showed consistent negative associations with UACR, UMALB, BUN, and sCr, and positive associations with eGFR. COR was inversely correlated with sCr and positively with eGFR. ALD was negatively correlated with UMALB and sCr

Spearman correlation analysis was performed to explore the associations between Urinary Stress Hormones levels and renal function indicators. The results showed that NE was negatively correlated with UACR, UMALB, BUN, and sCR, but positively correlated with eGFR **(**Fig. 4a-e**)**. Epinephrine displayed a negative correlation with UMALB and sCR, though no significant correlations were found with UACR, BUN, or eGFR. VMA showed negative correlations with UMALB and sCR, but no significant associations with UACR, BUN, or eGFR. HVA was inversely correlated with UMALB and sCR, but no significant correlations were found with other renal function indicators. 17-KS exhibited negative correlations with UACR, UMALB, BUN, and sCR, along with a positive correlation with eGFR**(**Fig. 4f-j**)**. 17-OHCS demonstrated negative correlations with BUN and sCR, but the correlation with UMALB was not statistically significant. COR displayed a negative correlation with sCR and a positive association with eGFR**(**Fig. 4k-o**)**. ALD exhibited negative correlations with both UMALB and sCR**(**Fig. 4p-t**)**. Detailed correlation coefficients and *P*-values are provided in Table 4**and** Fig. 4. Sex-stratified analyses revealed consistent overall trends, with NE demonstrating robust associations in both males and females. Specifically, in males, NE and 17-KS showed stronger correlations, whereas in females, in addition to NE, 17-OHCS, COR, and ALD exhibited stronger or additional associations. These additional results are provided in the Supplementary Materials (Tables S7 and S8).

**Table 4:** Correlation analysis between urinary stress hormone and renal function injury

Urinary stress hormones	UACR	UMALB	BUN	Cr	e-GFR
Norepinephrine	-0.194**	-0.288**	-0.306**	-0.522**	0.423**
Adrenaline	0.021	-0.165**	-0.001	-0.158**	-0.032
Homovanillic acid	0.031	-0.114**	-0.04	-0.197**	0.012
Vanillic mandelic acid	-0.046	-0.189**	-0.038	-0.196**	-0.055
17-ketosteroids	-0.165**	-0.207**	-0.104**	-0.162**	0.206**
17-hydroxysteroid	0.051	-0.01	-0.176**	-0.155**	0.206**
Cortisol	-0.105**	-0.175**	-0.172**	-0.306**	0.207**
Aldosterone	-0.087*	-0.205**	-0.158**	-0.301**	0.193**
β-hydroxybutyric acid	0.049	-0.052	-0.044	-0.243**	0.175**
Urine glucose	0.157**	0.117**	0.161**	-0.041	0.049
NAG	0.066	0.228**	0.151**	0.035	-0.011
Total protein	0.778**	0.860**	0.179**	0.265**	-0.204**
Retinol binding protein	0.233**	0.315**	0.316**	0.228**	-0.169**
Immunoglobulin G	0.662**	0.752**	0.284**	0.325**	-0.263**
Kappa light chain	0.583**	0.640**	0.165**	0.307**	-0.292**
Lambda light chain	0.771**	0.821**	0.211**	0.291**	-0.243**
Transferrin	0.412**	0.516**	0.326**	0.256**	-0.187**
α1-microglobulin	0.309**	0.298**	0.005	0.194**	-0.183**
β2-microglobulin	0.166**	0.218**	0.279**	0.268**	-0.240**

Note: at the level of 0.01 (double tailed), the correlation is significant. At 0.05 level (double tailed), the correlation was significant

### Univariate logistic regression analysis: risk factors associated with CKD with diabetes

**Fig. 5:**
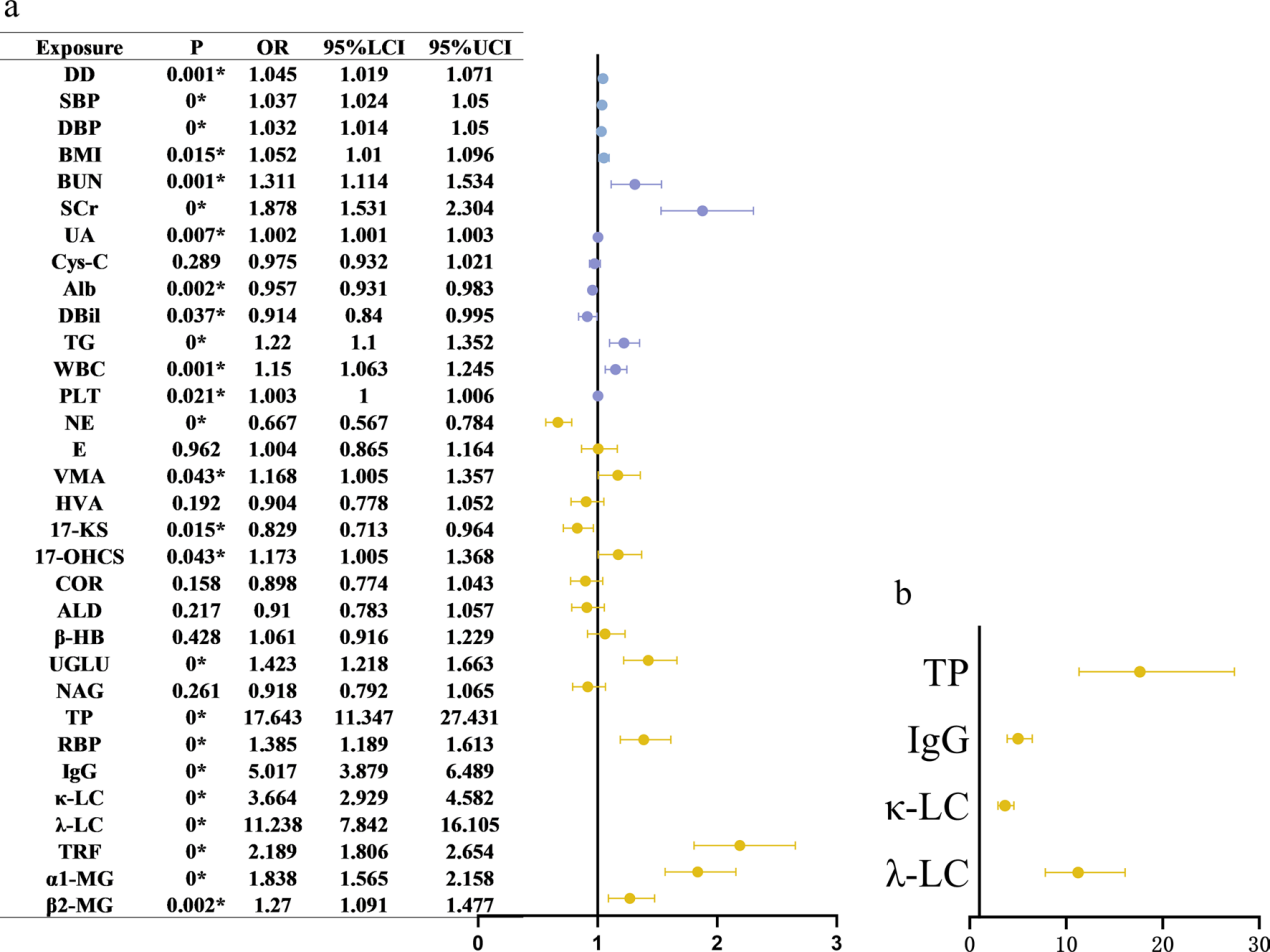
Predictors of diabetic kidney disease, results of univariate Logistic regression analysis. Forest plot illustrating odds ratios (ORs) and 95% confidence intervals (CIs) for the association between individual urinary adrenal hormone metabolites and the presence of CKD with diabetes. Each metabolite was analyzed separately using univariate logistic regression. Metabolite concentrations were log-transformed prior to analysis. An OR > 1 indicates that higher levels of the metabolite are associated with increased odds of CKD with diabetes, while an OR < 1 suggests a potential protective association. Several metabolites demonstrated statistically significant associations with CKD with diabetes, suggesting their potential relevance as biomarkers.*P* < 0.05 was considered statistically significant and is indicated by asterisks

Univariate logistic regression analysis showed that the following variables were associated with an increased risk of CKD with diabetes: longer disease duration, elevated systolic blood pressure, higher body mass index, elevated triglyceride levels, and higher serum concentrations of uric acid, BUN, and sCR. Regarding urinary adrenal hormones, NE was significantly inversely associated with CKD with diabetes, suggesting a potential protective effect. Conversely, VMA and UGLU emerged as significant risk factors for CKD with diabetes. Other hormonal indicators, including epinephrine, COR, and ALD were not significantly associated with CKD with diabetes. Additionally, several urinary proteins also exhibited significant correlations with CKD with diabetes risk. Detailed data are presented in Fig. 5a and b.In the sex-stratified univariate logistic regression analysis, urinary NE and 17-KS consistently emerged as protective factors, whereas 17-OHCS acted as a risk factor in the overall and male populations. Interestingly, these associations did not reach statistical significance in the female subgroup; however, NE, 17-KS, and 17-OHCS levels still tended to be higher in the CKD with diabetes group than in the DM group (Supplementary Tables S9–S10).

### Multivariate logistic regression analysis: predictive factors for CKD with diabetes

**Fig. 6:**
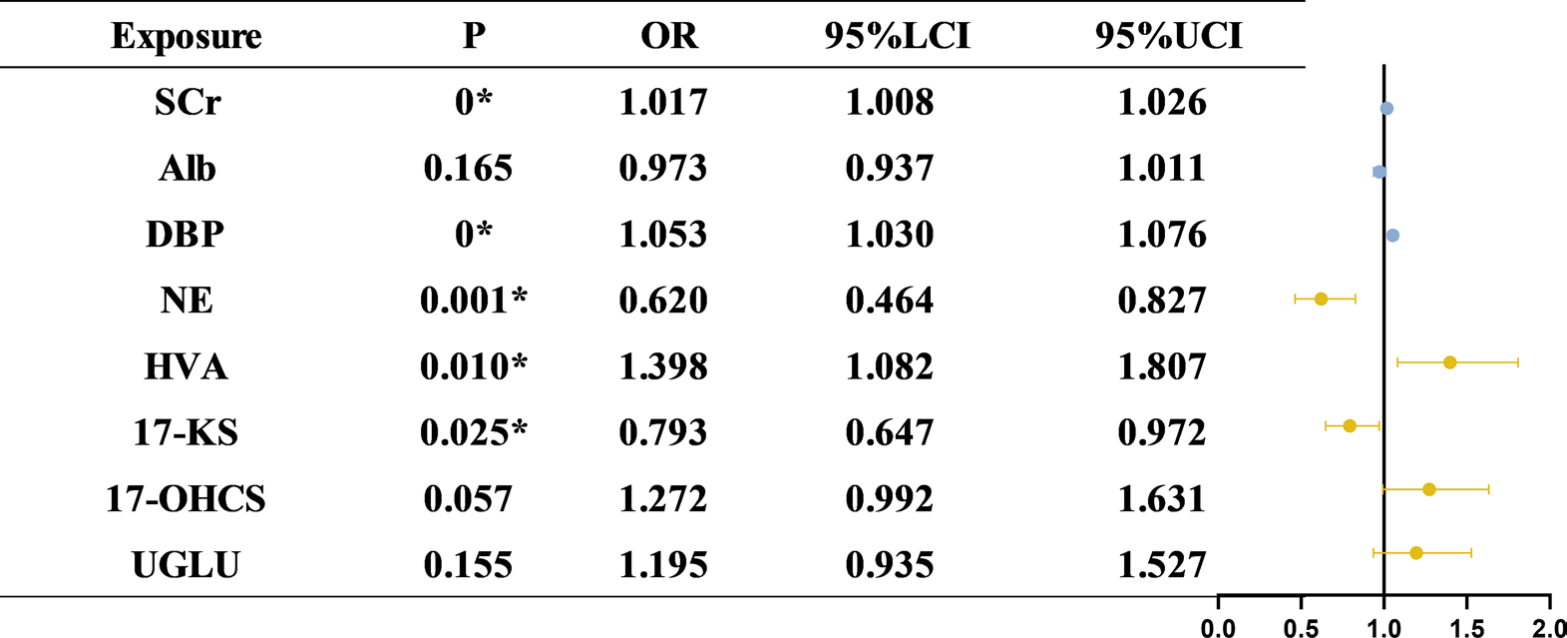
Predictors of diabetic kidney disease: Results of multivariate Logistic regression analysis. Forest plot presenting odds ratios (ORs) and 95% confidence intervals (CIs) from a multivariate logistic regression model evaluating the independent association between clinical indicators, urinary adrenal hormone metabolites, and CKD with diabetes. The model was adjusted for relevant covariates, including SCr, Alb, DBP, and others. Multivariate logistic regressionconfirmed the independent predictive value of NE, HVA, and 17-KS for CKD with diabetes after controlling for clinical covariates. Specifically, NE and 17-KS were negatively associated with CKD with diabetes risk (OR < 1), while HVA exhibited a positive association (OR > 1)

The results of multivariate logistic regression analysis identified SCr, DBP, and HVA as risk factors, while NE and 17-KS as protective factors for CKD with diabetes. SCr emerged as a strong risk factor, with each 1-unit increment associated with a 1.7% increase in CKD with diabetes risk. Similarly, DBP significantly contributes to CKD with diabetes susceptibility, with a 5.3% increase in risk per 1 mmHg elevation. HVA also showed a robust positive association with CKD with diabetes. Regarding protective factors, NE maintained a strong inverse relationship with CKD with diabetes, confirming its potential protective effect. In addition, 17-KS were inversely associated with CKD with diabetes risk, with a 20.7% risk reduction per unit increase. Although UGLU levels demonstrated a positive trend toward increased CKD with diabetes risk, the association was not statistically significant. Serum albumin also showed a negative correlation trend but did not reach significance. Further details are illustrated in Fig. 6. To further account for potential sex differences, sex-stratified multivariate logistic regression analyses were performed, urinary NE consistently acted as a protective factor across the overall, male, and female populations. Urinary 17-KS demonstrated a protective effect in the overall and female groups, but not in males. HVA emerged as a risk factor in the overall and female populations, whereas 17-OHCS was significant only in males. Notably, UGLU was identified as an additional risk factor in females (Supplementary Tables S11–S12).

### Diagnostic model performance for CKD with diabetes based on multivariate logistic regression

**Fig. 7:**
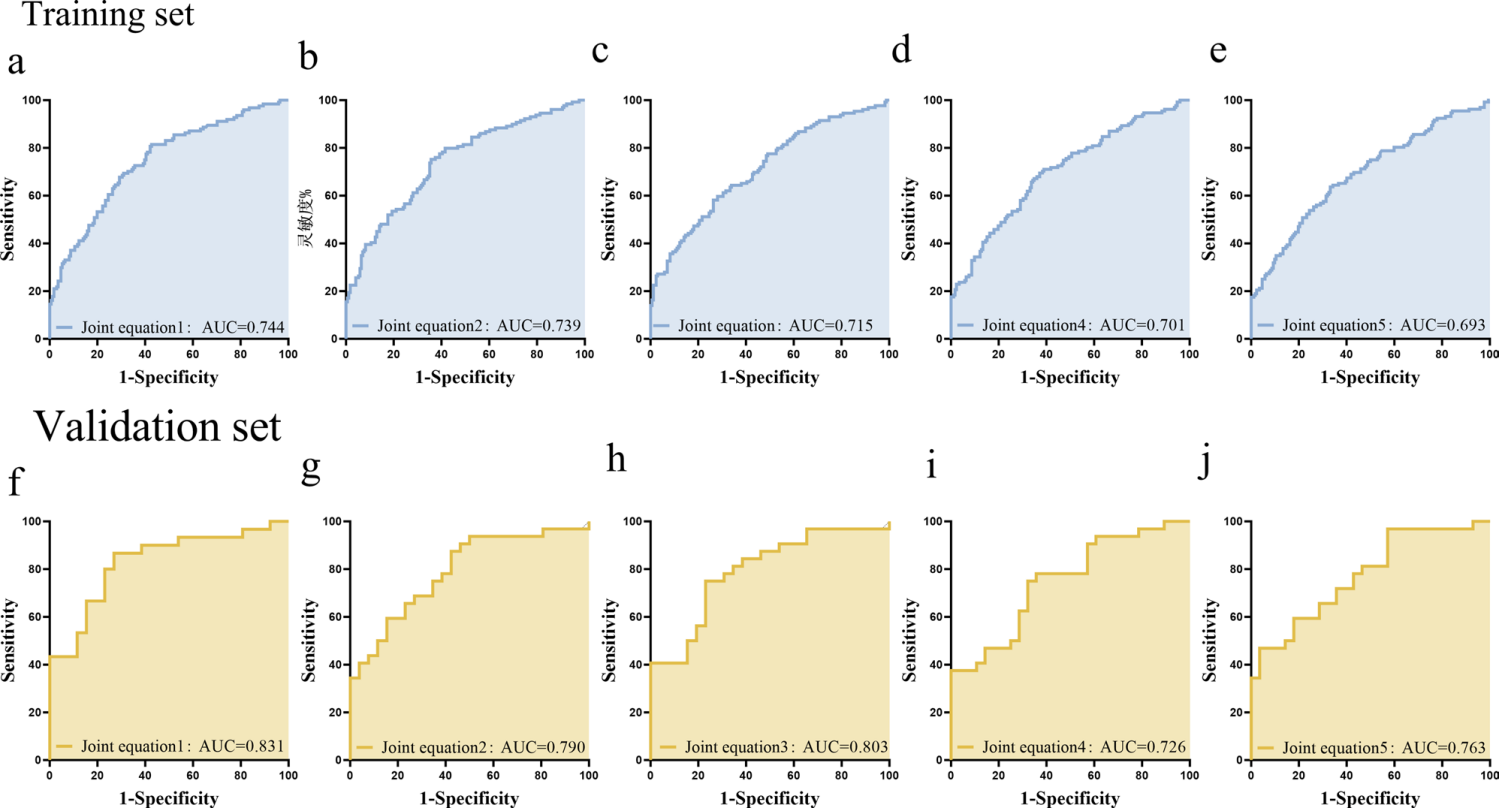
ROC curve and model evaluation. ROC curves show the discriminative ability of five multivariate logistic regression models (Joint Eqs. 1–5) constructed using combinations of clinical indicators and urinary adrenal hormone metabolites. The area under the ROC curve (AUC) was calculated to assess model performance, with higher AUCs indicating stronger predictive power for diabetic kidney disease (CKD with diabetes)

To assess the predictive utility of key risk factors and stress hormone biomarkers for CKD with diabetes, multivariate logistic regression models were developed. The performance of each model was evaluated using ROC curves and corresponding AUC values in both training and validation sets. Combined Eq. 1: Logit(P) = − 4.674 + (0.051 × DBP) + (0.017 × SCr) + (− 0.027 × ALb) + (− 0.479 × NE) + (0.335 × HVA) + (− 0.232 × 17-KS) + (0.241 × 17-OHCS) + (0.178 × UGLU). The AUC of the training set was 0.744, while that of the validation set was 0.831, indicating good classification performance, particularly in the validation cohort**(**Fig. 7a and f**)**. Combined Eq. 2: Logit(P) = − 4.545 + (0.054 × DBP) + (0.015 × SCr) + (− 0.031 × Alb) + (− 0.139 × 17-KS) + (− 0.499 × NE) + (0.392 × HVA). The AUC values of the training and validation sets were 0.739 and 0.790, respectively. This model maintained high predictive performance in both sets**(**Fig. 7b and g**)**. Combined Eq. 3: Logit(P) = − 3.865 + (0.050 × DBP) + (0.013 × SCr) + (− 0.034 × Alb) + (− 0.461 × NE). The AUCs were 0.715 and 0.803 in the training and validation sets, respectively, demonstrating improved predictive performance in validation**(**Fig. 7c and h**)**. Combined Eq. 4: Logit(P) = − 4.957 + (0.050 × DBP) + (0.021 × SCr) + (− 0.025 × ALb) + (0.313 × VMA). The AUC of the training set was 0.701, whereas that of the validation set was 0.726. While showing consistent results in both cohorts, this model yielded lower AUCs compared to the others**(**Fig. 7d and i**)**. Combined Eq. 5: Logit(P) = − 4.522 + (0.049 × DBP) + (0.019 × SCr) + (− 0.028 × ALb) + (− 0.146 × 17-KS). The AUCs of the training and validation sets were 0.693 and 0.763, respectively, indicating moderate diagnostic performance, with improvement in validation **(**Fig. 7e and j**)**.

## Discussion

In this study, urinary levels of several stress-related hormones, including NE, COR, ALD, 17-KS, 17-OHCS, and VMA, were markedly reduced in patients with CKD with diabetes compared to those diagnosed with diabetes alone. These hormonal changes were notably associated with various renal function indicators and metabolic parameters. Among them, NE demonstrated strong inverse correlations with conventional clinical markers of CKD with diabetes, suggesting its potential protective role in disease progression. Furthermore, the multivariate regression model integrating these urinary stress hormones exhibited robust discriminative performance, supporting their potential as noninvasive biomarkers for disease detection and risk stratification in CKD with diabetes.

### Potential protective role of reduced urinary catecholamines in CKD with diabetes

NE, a key neurotransmitter in the sympathetic nervous system [10], plays a vital role in regulating cardiovascular, metabolic, and immune functions under stress conditions [11]. Previous literature has shown that chronic hyperglycemia and insulin dysregulation in DM can lead to sustained sympathetic overactivity, which induces compensatory mechanisms, such as feedback inhibition, ultimately resulting in reduced NE levels [12]. Additionally, diabetic autonomic neuropathy, commonly observed in advanced CKD with diabetes, may further impair sympathetic outflow, possibly due to β-adrenergic receptor desensitization following chronic NE exposure. Another study found that ACEI or ARB may indirectly reduce sympathetic tone in CKD with diabetes patients by inhibiting the renin-angiotensin-aldosterone system (RAAS) pathways [13]. Our study suggests that alteration in sympathetic nervous system activity may contribute to the progression of CKD with diabetes. We also found that NE levels were negatively correlated with urinary albumin, BUN, and sCR.

Interestingly, NE levels were found to be higher in women, potentially reflecting cardiovascular protection mediated by sex hormones. While some studies have reported elevated NE concentrations in the elderly [14], we observed a decline in NE levels with advancing age. This discrepancy suggests that age-related changes in sympathetic activity may involve more complex, dynamic regulation [14]. A decline in NE levels may be associated with receptor desensitization or neurotransmitter depletion in the later stages of chronic overactivation. In univariate logistic regression, NE was significantly associated with a lower risk of CKD with diabetes(OR = 0.667), supporting its potential protective role in CKD with diabetes progression. Moreover, hyperglycemia has been shown to suppress catecholamine synthesis, further contributing to decreased NE levels in CKD with diabetes [15].

### Reactive decline in corticosteroid levels in CKD with diabetes patients

The negative correlation observed between COR and sCR suggests a potential association between reduced COR levels and renal impairment. However, some previous studies, such as that by Carine et al. [16], reported increased urinary COR levels in CKD with diabetes patients [16]. These inconsistencies may be attributed to differences in reference populations. Decreased renal responsiveness to glucocorticoids, along with oxidative stress and immune dysregulation, may lead to altered COR metabolism in CKD with diabetes [17]. Additionally, progressive renal impairment can disrupt COR clearance and feedback regulation, resulting in decreased circulating and urinary levels [18]. Hyperglycemia itself has also been implicated in suppressing glucocorticoid secretion [19]. These mechanisms highlight a need for further molecular and mechanistic studies to elucidate the role of COR in CKD with diabetes.

The decline in ALD levels may reflect feedback inhibition resulting from the overactivation of the RAAS. ALD has been shown to promote renal inflammation and fibrosis, two key processes in CKD with diabetes progression, via mineralocorticoid receptors [20, 21]. RAAS inhibitors (e.g. ACEI and ARB), which are taken by 37% of CKD with diabetes patients and 11.6% of DM patients, may reduce angiotensin II activity and subsequently lower ALD levels, thereby decreasing intraglomerular pressure and proteinuria [22, 23], particularly in patients with CKD with diabetes receiving RAAS blockade therapy [24]. Thus, the reduced ALD levels observed in the CKD with diabetes group may be related to RAAS blockade. Although ALD holds potential as a CKD with diabetes biomarker, the confounding effect of pharmacological treatment must be considered. Further studies are needed to clarify the diagnostic and prognostic value of ALD in CKD with diabetes.

### Application value and suitability of urinary stress hormones in diagnosis of CKD with diabetes

Our study constructed multivariate logistic regression models that integrated both conventional clinical risk factors, such as blood pressure and metabolic parameters, and novel urinary adrenal hormone biomarkers, including NE, 17-KS, 17-OHCS, and VMA. This approach reflects the multifactorial nature of CKD with diabetes pathogenesis while highlighting the diagnostic value of stress hormone profiles. Among the five models evaluated, Eq. 1 incorporated the most comprehensive set of variables. Preprocessing techniques, such as logarithmic transformation, were applied to optimize variable distributions and enhance model robustness. This equation achieved the highest discriminatory power, with an AUC of 0.831 in the validation set, suggesting excellent predictive performance and generalizability to external datasets. However, the increased complexity of Eq. 1 poses potential challenges, including risks of overfitting, multicollinearity, and higher laboratory testing costs. Simplified models, although demonstrating slightly lower AUC values, still maintained satisfactory diagnostic performance in the validation set. Equation 3 effectively balances clinical feasibility with predictive power. With its reduced number of variables, stable validation performance, and straightforward clinical interpretation, Eq. 3 offers a reliable model for the diagnosis of CKD with diabetes. These findings underscore the significance of NE as a valuable diagnostic and predictive tool.

### Limitations

Sex-stratified analysis revealed both robustness and heterogeneity of urinary hormone biomarkers in CKD with diabetes. Correlation and regression results showed that urinary NE and 17-KS acted as protective factors in the overall and male populations, whereas 17-OHCS emerged as a risk factor only in males; these associations did not reach statistical significance in females. In multivariate models, urinary NE consistently retained a protective role across overall, male, and female groups; 17-KS remained protective in the overall and female populations but was not significant in males. Conversely, HVA emerged as a risk factor in the overall and female populations, while 17-OHCS was significant only in males. Notably, such sex-specific differences may be related to endocrine and physiological bases: women generally exhibit lower levels of adrenal cortical and sympathetic hormones and are more strongly regulated by estrogen. Beyond its metabolic effects, estrogen exerts antioxidant, anti-inflammatory, and vasoprotective actions, which may partly substitute for the protective roles of NE and 17-KS, thereby “masking” their statistical associations with CKD with diabetes risk in females. These findings highlight the importance of considering sex background when interpreting the renal protective or deleterious roles of stress hormones and underscore the need for sex-stratified validation cohorts in future studies to yield clinically meaningful insights.A brief discussion on T2DM subtype heterogeneity is provided in the Supplementary Discussion.

In conclusion, the integration of novel urinary adrenal stress hormone markers, particularly NE, 17-KS, and HVA, into logistic regression models improved diagnostic performance for CKD with diabetes in both training and validation cohorts. These findings suggest that combining conventional clinical indicators with stress-related hormonal biomarkers can enhance diagnostic accuracy and provide complementary information for disease evaluation. Moreover, the distinct metabolic signatures reflected by these urinary hormones may offer novel insights into the neuroendocrine and metabolic mechanisms underlying CKD with diabetes.

Given the cross-sectional design of this study, prospective longitudinal studies are warranted to further validate the diagnostic and prognostic value of these urinary biomarkers and to evaluate their potential clinical utility in disease assessment and management. Moreover, larger multicenter studies are needed to confirm the stability and generalizability of the models across diverse populations.Another methodological limitation is that urinary hormone concentrations were normalized using urinary creatinine levels, which may be affected by reduced renal excretory function in CKD with diabetes. Although this approach is commonly used to correct for urine dilution, differences in kidney performance between diabetic and CKD populations could introduce variability. Future studies should include urine osmolarity or specific gravity measurements to validate and refine normalization strategies for urinary stress hormone analysis.In addition, although mass spectrometry provides higher analytical sensitivity and specificity, it was not employed in this study due to its high cost and limited feasibility for large-scale population screening. Instead, homogeneous enzyme immunoassay and latex-enhanced immunoturbidimetry were selected for their robustness, reproducibility, and suitability for automated high-throughput clinical applications, aligning with the translational goal of this work.

## Supplementary Information

Below is the link to the electronic supplementary material.

Supplementary Material 1

## Data Availability

The datasets used and analysed during the current study are available from the corresponding author on reasonable request.
