# Supplementary material for: Urinary stress hormones and their metabolites as predictive biomarkers for CKD with diabetes
Source: BMC Nephrol. 2025 Dec 24;27:75. doi: 10.1186/s12882-025-04717-9 (PMC12849337; doi:10.1186/s12882-025-04717-9)
Supplement: Supplementary file 1 — Supplementary Material 1 [file 12882_2025_4717_MOESM1_ESM.docx]

**Supplementary materials**

**Methods**

**1. Data Collection**

General clinical data were collected from the electronic medical record system of The First Affiliated Hospital Of Wannan Medical College.

1.1 Demographic and basic statistical data includes patient name, sex, age (years), height (cm), weight (kg), admission systolic blood pressure (SBP, mmHg), diastolic blood pressure (DBP, mmHg), diabetes duration (years), smoking history, and bleeding history.

1.2 Medical history includes comorbidities such as hypertension, coronary atherosclerotic heart disease, stroke, ischemic cardiomyopathy, and heart failure.

1.3 Medication history includes Antihypertensive drugs: ACEI/ARB, CCB, etc.Antidiabetic drugs: ①Oral hypoglycemic agents: biguanides, SGLT-2 inhibitors.；②Injectable insulin.

1. **Laboratory Indicators**

**2.1 Blood Biochemical Tests**

Patients fasted for at least 8 hours. Venous blood and urine samples were collected the following morning. Blood tests were performed using the SIEMENS ADVIA WorkCell biochemical-immunoassay assembly line. Collected parameters included: Fasting plasma glucose (FPG), serum creatinine (Scr, μmol/L), blood urea nitrogen (BUN, mmol/L), serum uric acid (SUA, μmol/L), cystatin C (Cys-C), total cholesterol (TC, mmol/L), triglycerides (TG, mmol/L), HDL (mmol/L), LDL (mmol/L), glycated hemoglobin (HbA1c, %), hemoglobin (Hb, g/L), platelet count (PLT), total protein (TP), albumin (Alb, g/L), alanine aminotransferase (ALT), aspartate aminotransferase (AST), estimated glomerular filtration rate (eGFR, calculated using the CKD-EPI formula), urinary protein (qualitative and quantitative), urinary albumin (mg/L), urinary creatinine (g/L), and albumin-to-creatinine ratio (ACR). HbA1c was measured via liquid chromatography (Lange Technology AH-600 analyzer).

**2.2 Urine Collection and Analysis**

**Table S1. Assay methods for urinary hormones and metabolites**

| Biomarker | Method | Catalog Number | Manufacturer |
| --- | --- | --- | --- |
| NE | Latex-enhanced immunoturbidimetry | NL0201 | Changsha Boyuan Medical Technology,Co., Ltd., China |
| E | Latex-enhanced immunoturbidimetry | ML0601 | Changsha Boyuan Medical Technology, Co., Ltd.,China |
| VMA | Homogeneous enzyme immunoassay | HH0701 | Suzhou Boyuan Medical Technology,Co., Ltd., China |
| HVA | Homogeneous enzyme immunoassay | VH0210 | Changsha Boyuan Medical Technology,Co., Ltd., China |
| 17-KS | Homogeneous enzyme immunoassay | KH0110 | Suzhou Boyuan Medical Technology,Co., Ltd., China |
| 17-OHCS | Homogeneous enzyme immunoassay | OH0110 | Suzhou Boyuan Medical Technology,Co., Ltd., China |
| COR | Homogeneous enzyme immunoassay | HH0102 | Suzhou Boyuan Medical Technology,Co., Ltd., China |
| ALD | Homogeneous enzyme immunoassay | AH0101 | Suzhou Boyuan Medical Technology,Co., Ltd., China |
| Urinary creatinine | Homogeneous enzyme immunoassay | CH0301 | Suzhou Boyuan Medical Technology,Co., Ltd., China |

**Note：**This table provides detailed information on the measurement methods, reagent specifications, and manufacturers for urinary hormones and metabolites analyzed in this study.

**3.Formula and Grouping Methods**

BMI compute and grading groups is: BMI= weight（kg）/[ height（m）2]，BMI<18.5kg/m2 is underweight，18.5kg/m2≤BMI<24kg/m2 is proper，24kg/m2≤BMI<28kg/m2 is overweight，BMI≥28kg/m2 is obesity.

2021 CKD-EPI Equation for eGFR:

| Table 2021 CKD-EPI Calculation formula | | |
| --- | --- | --- |
| Sex | Scr（umol/L） | eGFR calculation formula |
| female | ≤61.9umol/L | 142×（Scr/61.88）^-0.241^×0.9938^age^×1.012 |
|  | ＞61.9umol/L | 142×（Scr/61.88）^-1.200^×0.9938^age^×1.012 |
| male | ≤79.6umol/L | 142×（Scr/79.56）^-0.302^×0.9938^age^ |
|  | ＞79.6umol/L | 142×（Scr/79.56）^-1.200^×0.9938^age^ |

**4.Grouping Criteria**

Participants were stratified by sex, age, diabetes duration, BMI, UACR:（1）Sex: Male and female. (2) Age: <40, 40–60, ≥60 years.(3)Diabetes duration: ≤5, 6–10, >10 years.(4) BMI: <18.5, 18.5–23.9, 24–27.9, ≥28 kg/m².(5)UACR: Normal (UACR <30 mg/g), microalbuminuria (30 ≤ UACR <300 mg/g), macroalbuminuria (UACR ≥300 mg/g).

**Results**

**1.Two groups of patients' baseline characteristics**

In terms of past medical history, the history of hypertension was 34.4% in the diabetes group and 62.1% in the diabetic kidney disease group. The proportion of patients with a smoking history in the diabetes group was 20.1%, and in the diabetic kidney disease group, it was 12.1%. For cardiovascular events, there were 32 people in the diabetes group and 12 people in the diabetic kidney disease group. Regarding the history of cerebrovascular disease, there were 20 people in the diabetes group and 10 people in the diabetic kidney disease group. In terms of the past history of combined tumors, there were 13 people in the diabetes group and 8 people in the diabetic kidney disease group. Regarding the main concomitant medications, the proportion of using aspirin in the diabetic kidney disease group was 10%, and in the diabetes group, it was 3.8% ; for atorvastatin use, the proportion in the diabetic kidney disease group was 21.4%, and in the diabetes group, it was 14.3%; the proportion of insulin use in the diabetic kidney disease group was 39.9%, higher than 31.1% in the diabetes group; the proportion of dapagliflozin use in the diabetic kidney disease group was 53.7%, and in the diabetes group, it was 34.5% ; the proportion of ACEI/ARB use in the diabetic kidney disease group was 37%, and in the diabetes group, it was 11.6%; the proportion of clopidogrel use in the diabetes group and the diabetic kidney disease group was 2.9% and 2.8%, respectively; the proportion of metformin use in the diabetes group and the diabetic kidney disease group was 53.9% and 48.4%, respectively. **See Table S2 for details.**

In terms of urine routine, for urine glucose, in the diabetes group, 132 cases were negative, 15 cases were 1+, 11 cases (3.7%) were 2+, 78 cases were 3+, and 64 cases were 4+. In the diabetic kidney disease group, 53 cases were negative, 11 cases were 1+, 17 cases were 2+, 68 cases were 3+, and 63 cases were 4+; for urine ketone bodies, in the diabetes group, 282 cases were negative, 11 cases were 1+, 12 cases were 2+, and 2 cases were 3+. In the diabetic kidney disease group, 180 cases were negative, 2 cases were 1+, 25 cases were 2+, and 13 cases were 3+; for urine protein, in the diabetes group, 271 cases were negative, 28 cases were 1+, 6 cases were 2+, and 1 case was 3+. In the diabetic kidney disease group, 117 cases were negative, 36 cases were 1+, 39 cases were 2+, and 25 cases were 3+. **See Table S2 for details.**

**Table S2. Patients with Diabetes and CKD with diabetes: General and Clinical Characteristics**

| Baseline Characteristics |  | DM（n = 449） | CKD with diabetes（n = 286） | z/ χ^2^ | P Vaule |
| --- | --- | --- | --- | --- | --- |
|  |  |  |  |  |  |
|  | Hypertension History | 111（46.8%） | 155（78.7%） | 45.98 | 0* |
|  | Smoking History | 73(20.1%) | 28(12.1%) | 6.31 | 0.012* |
|  | Cardiovascular Events | 32(9%) | 12(5.2%) | 2.92 | 0.087 |
|  | Cerebrovascular Events | 20(6.8%) | 10(4.5%) | 1.23 | 0.267 |
|  | Tumor History | 13(4.7%) | 8(3.7%) | 0.34 | 0.562 |
| Past Medical History | Aspirin | 17（3.8%） | 28（10%） | 11.29 | 0.001* |
|  | Clopidogrel | 13（2.9%） | 8（2.8%） | 0.002 | 0.962 |
|  | Atorvastatin | 64（14.3%） | 60（21.4%） | 6.04 | 0.014* |
|  | Insulin | 139（31.1%） | 112（39.9%） | 5.86 | 0.015* |
|  | Dapagliflozin | 154（34.5%） | 151（53.7%) | 26.36 | 0* |
|  | Metformin | 241(53.9%) | 136(48.4%) | 2.1 | 0.147 |
|  | ACEI/ARB | 52(11.6%) | 104(37%) | 66 | 0* |
|  | CCB | 45(10%) | 84(29.4%) | 45.2 | 0* |
| Urinalysis | Urine Glucose |  |  | 21.85 | 0* |
|  | Negative | 132 (44%) | 53 (25%) |  |  |
|  | 1+ | 15 (5%) | 11 (5.2%) |  |  |
|  | 2+ | 11 (3.7%) | 17 (8%) |  |  |
|  | 3+ | 78 (26%) | 68 (32.1%) |  |  |
|  | 4+ | 64 (21.3%)） | 63 (29.7%) | 27.78 | 0* |
|  | Urine Ketone Bodies |  |  |  |  |
|  | Negative | 282 (91.9%) | 180 (81.8%) |  |  |
|  | 1+ | 11 (3.6%) | 2 (0.9%) |  |  |
|  | 2+ | 12 (3.9%) | 25 (11.4%) |  |  |
|  | 3+ | 2 (0.7%) | 13 (5.9%) |  |  |
|  | Urine Protein |  |  | 97.45 | 0* |
|  | Negative | 271 (88.6%) | 117 (53.7%) |  |  |
|  | 1+ | 28 (9.2%) | 36 (16.5%) |  |  |
|  | 2+ | 6 (2%) | 39 (17.9%) |  |  |
|  | 3+ | 1 (0.3%) | 25 (11.5%) |  |  |
|  | 4+ | 0 (0%) | 1 (0.5%) |  |  |
|  | Urine White Blood Cell |  |  | 5.69 | 0.155 |
|  | Negative | 146 (82%) | 66 (75.9%) |  |  |
|  | 1+ | 20 (11.2%) | 9 (10.3%) |  |  |
|  | 2+ | 7 (3.9%) | 4 (4.6%) |  |  |
|  | 3+ | 5 (2.8%) | 8 (9.2%) |  |  |

**Note：***indicates P < 0.05 ，with statistically significant differences.

**2.Comparative Analysis of Urinary Stress Hormones under Different Gender Stratifications**

The results of the gender stratification analysis show that there are significant differences in multiple metabolic indicators between men and women. Among the catecholamine hormones, the concentrations of norepinephrine [225.90 (178.93, 282.86)], epinephrine [68.94 (55.15, 89.17)], homovanillic acid [5.48 (4.07, 7.71)], and vanillylmandelic acid [7.17 (5.52, 8.80)] in the female group are significantly higher than those in the male group (all P < 0.001). Among them, norepinephrine is the highest in women (Z = -8.49, P < 0.001), while the corresponding levels in the male group are 179.03, 60.55, 4.58, and 5.35, respectively. Among the glucocorticoids and mineralocorticoids, cortisol [311.53 (213.69, 426.36)] and aldosterone [8.54 (6.65, 11.24)] in the female group are also significantly higher than those in the male group (P = 0.005 and P < 0.05). **See Table S3.**

**Table S3. Camparative analysis of urinary stress hormones under different gender stratifications**

| Urinary stress hormones | Male | Female | Z | P |
| --- | --- | --- | --- | --- |
| Norepinephrine | 179.03 (143.96, 220.43) | 225.90 (178.93, 282.86) | -8.49 | 0* |
| Epinephrine | 60.55（48.74,89.17） | 68.94（55.15,89.17） | -4.40 | 0* |
| Homovanillic acid | 4.58（3.49,6.21） | 5.48（4.07,7.71） | -5.23 | 0* |
| Vanillylmandelic acid | 5.35（4.07,6.54） | 7.17（5.52,8.80） | -10.09 | 0* |
| 17-ketosteroid | 6.35 (4.65, 8.51) | 5.58 (4.13, 7.79) | -3.44 | 0.001* |
| 17-hydroxy steriod | 5.40（4.29,7.31） | 5.02（3.72） | -2.86 | 0.004* |
| Cortisol | 279.01 (189.92, 372.83) | 311.53 (213.69, 426.36) | -2.83 | 0.005* |
| Aldosterone | 7.57（5.75,9.97） | 8.54（6.65,11.24） | -3.96 | 0* |
| β-Hydroxybutyric acid | 16.24（11.55,28.11） | 18.49（13.22,25.88） | -1.96 | 0.050* |
| Urinary glucose | 34.55 (2.15, 165.65) | 17.11 (0.80, 153.12) | -2.28 | 0.023* |
| NAG | 15.92 (7.29, 27.00) | 13.00 (6.75, 24.00) | -2.08 | 0.038* |
| Total protein | 10.30 (6.10, 20.40) | 8.60 (5.70, 18.43) | -1.77 | 0.077 |
| Retinol blinding protein | 3.91 (0.40, 10.23) | 4.29 (0.41, 8.72) | -1.16 | 0.245 |
| Immunoglobulin G | 9.60 (5.71, 20.04) | 7.64 (4.42, 17.35) | -2.51 | 0.012* |
| Kappa light chain | 5.67 (2.36, 13.10) | 3.38 (1.50, 9.43) | -3.66 | 0* |
| Lambda light chain | 2.00 (0.77, 5.83) | 1.29 (0.56, 4.81) | -2.15 | 0.031* |
| Transferrin TRF | 12.15 (2.60, 22.77) | 9.10 (2.57, 18.71) | -2.2 | 0.028* |
| α1 Microglobulin | 0.76 (0.09, 8.69) | 0.26 (0.05, 4.45) | -3.23 | 0.001* |
| β2 Microglobulin | 0.24 (0.10, 0.61) | 0.17 (0.08, 0.39) | -3.56 | 0* |

**Note：***indicates P < 0.05 ，with statistically significant differences.

**3.The differences in urinary stress hormones at different disease course stratifications**

This study conducted stratified analysis of urinary metabolites based on disease duration (≤5 years, 6-10 years, >10 years) to explore whether there were significant changes in urinary metabolites as the disease progressed, and to evaluate their potential role in the progression of diabetes and the risk of diabetic kidney disease (**CKD with diabetes**). Urinary glucose (P = 0.001, H = 13.51) showed significant differences among the three groups (P < 0.05). The values were 7.55 (0.90, 150.48) in the ≤5-year group, 53.92 (2.26, 155.17) in the 6-10-year group, and 47.60 (2.21, 165.88) in the >10-year group. Urinary glucose was significantly higher in the 6-10-year and >10-year groups compared to the ≤5-year group. However, other metabolites, such as norepinephrine (P = 0.842), epinephrine (P = 0.925), homovanillic acid (P = 0.968), vanillylmandelic acid (P = 0.697), 17-ketosteroids (P = 0.749), 17-hydroxysteroids (P = 0.498), cortisol (P = 0.112), aldosterone (P = 0.870), NAG (P = 0.597), β-hydroxybutyric acid (P = 0.169), total protein (P = 0.391), retinol binding protein (P = 0.314), immunoglobulin G (P = 0.860), Kappa light chain (P = 0.811), Lambda light chain (P = 0.403), transferrin (P = 0.666), α1-microglobulin (P = 0.949), and β2-microglobulin (P = 0.239), did not show significant differences among the different disease duration groups. **See Table S4.**

**Table S4. The differences in urinary stress hormones at different disease course stratifications**

| Urinary stress hormones | ≤ 5 years | 6-10 years | >10 years | H | P |
| --- | --- | --- | --- | --- | --- |
| norepinephrine | 209.83 (168.40, 255.64) | 189.60 (149.48, 244.23) | 178.87 (143.10, 247.96) | 0.35 | 0.842 |
| epinephrine | 60.29（48.30,82.72） | 66.65（54.49,84.90） | 67.37（52.08,91.16） | 0.16 | 0.925 |
| homovanillic acid | 4.68（3.47,6.48） | 5.11（3.77,6.96） | 5.34（4.08,7.48） | 0.07 | 0.968 |
| vanillylmandelicious acid | 5.86（4.60,7.55） | 5.62（4.46,7.33） | 5.98（4.97,8.10） | 0.72 | 0.697 |
| 17-ketosteroids | 6.38 (4.43, 8.47) | 5.60 (4.51, 8.07) | 5.53 (3.86, 7.37) | 0.58 | 0.749 |
| 17-hydroxysteroids | 5.30（4.23,7.37） | 4.99（3.73,6.65） | 5.23（3.92,7.22） | 1.39 | 0.498 |
| cortisol | 307.98 (201.71, 430.57) | 264.83 (191.63, 400.16) | 280.78 (177.72, 363.66) | 4.38 | 0.112 |
| aldosterone | 8.46（6.00,11.08） | 7.19（5.77,10.62） | 7.98（6.39,10.06） | 0.28 | 0.870 |
| β-hydroxybutyric acid | 16.82（11.95,26.98） | 17.19（12.48,29.23） | 19.44（12.75,27.96） | 3.55 | 0.169 |
| Urinary glucose | 7.55 (0.90, 150.48) | 53.92 (2.26, 155.17) | 47.60 (2.21, 165.88) | 13.51 | 0.001* |
| NAG | 14.14 (7.42, 25.00) | 14.00 (6.79, 26.00) | 13.00 (5.63, 23.20) | 1.03 | 0.597 |
| total protein | 8.80 (5.60, 15.20) | 9.90 (6.20, 20.95) | 11.40 (6.00, 41.10) | 1.88 | 0.391 |
| retinol binding protein | 4.42 (0.41, 9.09) | 4.20 (0.43, 9.88) | 3.34 (0.35, 8.51) | 2.32 | 0.314 |
| immunoglobulin G | 7.46 (4.56, 12.60) | 9.92 (5.23, 21.28) | 10.93 (5.91, 28.53) | 0.30 | 0.860 |
| Kappa light chain | 3.34 (1.49, 8.08) | 5.64 (2.31, 12.52) | 6.49 (2.43, 18.99) | 0.42 | 0.811 |
| Lambda light chain | 1.28 (0.54, 3.40) | 1.98 (0.62, 6.69) | 2.56 (0.81, 10.09) | 1.82 | 0.403 |
| transferrin | 9.10 (2.23, 18.08) | 11.17 (1.60, 23.64) | 12.26 (2.33, 23.16) | 0.81 | 0.666 |
| α1-microglobulin | 0.26 (0.06, 4.48) | 1.23 (0.12, 8.40) | 2.14 (0.08, 9.99) | 0.11 | 0.949 |
| β2-microglobulin | 0.21 (0.10, 0.44) | 0.23 (0.09, 0.65) | 0.22 (0.08, 0.68) | 2.87 | 0.239 |

Note: * indicates P < 0.05, which is statistically significant.

**4.The differences in urinary stress hormones at different age**

Specifically, the concentration of norepinephrine was 211.60 (182.24, 263.32) in the year-old group and 195.08 (152.36, 245.74) in the ≥60-year-old group (P = 0.005, H = 10.67). The concentration of epinephrine increased significantly with age (P < 0.001, H = 49.35), rising from 52.94 in the year-old group to 69.03 in the ≥60-year-old group. Homovanillic acid (P < 0.001) and vanillylmandelic acid (P < 0.001) also showed a similar trend. Additionally, the concentrations of 17-ketosteroids (P = 0.002) and 17-hydroxysteroids (P = 0.012) decreased with age. The concentration of kappa light chain increased significantly with age (P = 0.001), with the highest level in the ≥60-year-old group at 5.53 (2.33, 12.57). There were no statistically significant differences in the remaining metabolic indicators among different age groups, including cortisol (P = 0.097), aldosterone (P = 0.062), β-hydroxybutyric acid (P = 0.263), urine glucose (P = 0.098), NAG (P = 0.958), α1-microglobulin (P = 0.090), total protein (P = 0.835), retinol binding protein (P = 0.623), immunoglobulin G (P = 0.346), lambda light chain (P = 0.333), transferrin (P = 0.831), and β2-microglobulin (P = 0.287). See **Table S5**.

**Table S5. The differences in urinary stress hormones at different age**

| Urinary stress hormones | <40-year-old | ≥40-year-old，<60-year-old | ≥ 60-year-old | H | P |
| --- | --- | --- | --- | --- | --- |
| norepinephrine | 211.60 (182.24, 263.32) | 187.50 (154.76, 244.14) | 195.08 (152.36, 245.74) | 10.67 | 0.005* |
| epinephrine | 52.94（41.55,65.67） | 59.84（49.89,78.47） | 69.03（57.12,91.66） | 49.35 | 0* |
| homovanillic acid | 3.78（3.00,5.18） | 4.64（3.50,6.40） | 5.44(4.33,7.67) | 49.63 | 0* |
| vanillylmandelicious acid | 4.83(3.64,6.04) | 5.49(4.47,7.12) | 6.71(5.34,8.59) | 67.49 | 0* |
| 17-ketosteroids | 7.17 (4.96, 9.93) | 6.16 (4.38, 8.14) | 5.55 (4.38, 7.65) | 12.55 | 0.002* |
| 17-hydroxysteroids | 5.65(4.08,8.50) | 5.23(4.19,7.38) | 5.12(3.88,6.37) | 8.84 | 0.012* |
| cortisol | 296.99 (176.45, 428.88) | 285.15 (188.58, 373.85) | 302.47 (211.20, 405.53) | 4.67 | 0.097 |
| aldosterone | 8.22(5.46,12.55) | 7.57(5.75,10.15) | 8.13(6.56,10.58) | 5.57 | 0.062 |
| β-hydroxybutyric acid | 19.46(11.47,55.43) | 16.25(11.53,28.48) | 17.32(13.15,25.19) | 2.67 | 0.263 |
| Urinary glucose | 7.87 (1.04, 129.56) | 44.31 (1.23, 181.10) | 26.75 (1.02, 145.84) | 4.65 | 0.098 |
| NAG | 13.00 (7.00, 26.00) | 15.00 (7.34, 24.00) | 14.86 (6.52, 26.79) | 0.09 | 0.958 |
| total protein | 9.10 (5.58, 26.55) | 9.70 (5.90, 18.30) | 9.80 (5.83, 19.60) | 0.36 | 0.835 |
| retinol binding protein | 4.72 (0.46, 8.59) | 3.93 (0.37, 9.54) | 4.23 (0.44, 10.30) | 0.95 | 0.623 |
| immunoglobulin G | 7.98 (4.42, 17.45) | 8.48 (5.15, 18.96) | 9.41 (5.11, 20.80) | 2.13 | 0.346 |
| Kappa light chain | 2.98 (1.23, 8.04) | 4.44 (1.81, 11.01) | 5.53 (2.33, 12.57) | 13.26 | 0.001* |
| Lambda light chain | 1.53 (0.45, 5.75) | 1.61 (0.66, 4.92) | 1.97 (0.70, 5.79) | 2.2 | 0.333 |
| transferrin | 10.64 (3.88, 17.10) | 10.75 (2.57, 20.05) | 10.39 (1.79, 22.87) | 0.37 | 0.831 |
| α1-microglobulin | 0.18 (0.06, 3.65) | 0.48 (0.07, 7.07) | 0.76 (0.06, 8.34) | 4.81 | 0.090 |
| β2-microglobulin | 0.18 (0.10, 0.44) | 0.20 (0.08, 0.48) | 0.24 (0.10, 0.67) | 2.5 | 0.287 |

Note: * indicates P < 0.05, which is statistically significant.

**5. Differences in urinary stress hormones under different BMI stratification**

In this study, we analyzed the changes in urinary metabolites by BMI group (<18.5, 18.5-23.9, 24-27.9, and ≥28 kg/m²). The results revealed notable variations in several metabolic indicators across BMI groups. Among them, epinephrine concentration exhibited a clear decline with increasing BMI (P < 0.001, H = 19.99), dropping from 84.77 in the BMI <18.5 group to 57.76 in the BMI ≥28 group. Homovanillic acid levels also displayed marked differences between groups (P = 0.003), while the variation in vanillylmandelic acid approached statistical significance (P = 0.066). The level of 17-ketosteroids progressively decreased with higher BMI (P = 0.030), whereas 17-hydroxysteroids remained relatively stable (P = 0.763). Both cortisol (P = 0.004) and aldosterone (P < 0.001) concentrations showed a downward trend with increasing BMI. Norepinephrine, however, did not demonstrate a statistically meaningful difference between groups (P = 0.067). Among the protein-related metrics, total protein concentration was significantly elevated with higher BMI (P = 0.001), and Lambda light chain (P = 0.001) and β2-microglobulin (P = 0.004) also exhibited significant variations. In contrast, NAG, β-hydroxybutyrate, urinary glucose, retinol-binding protein, immunoglobulin G, Kappa light chain, transferrin, and α1-microglobulin showed no statistically significant differences across BMI groups (P > 0.05).see**Table S6**

**Table S6. Differences in Urinary Stress Hormones by BMI Stratification**

| Urinary stress hormones | BMI< 18.5 | 18.5 ≤ BMI < 23.9 | 24 ≤ BMI < 27.9 | BMI ≥ 28 | H | P |
| --- | --- | --- | --- | --- | --- | --- |
| norepinephrine | 221.04 (187.13, 275.91) | 199.06 (164.60, 250.49) | 188.48 (143.99, 238.43) | 230.07 (174.75, 265.66) | 7.16 | 0.067 |
| adrenaline | 84.77(62.22,117.95) | 66.19(52.08,87.14) | 59.78(48.30,72.54) | 57.76(43.37,73.67) | 19.99 | 0* |
| Homovanillic acid | 4.01(2.99,8.46) | 5.31(4.14,7.07) | 4.51(3.47,6.44) | 4.33(3.15,5.94) | 13.80 | 0.003* |
| Vanilla Mandelic Acid | 5.98(4.14,8.08) | 5.91(4.88,7.57) | 5.36(4.14,7.07) | 5.70(4.65,7.78) | 7.19 | 0.066 |
| 17 Ketosteroids | 7.05 (4.78, 9.87) | 6.13 (4.64, 8.42) | 5.60 (4.01, 7.61) | 4.92 (3.52, 7.14) | 8.97 | 0.030* |
| 17 hydroxysteroids | 5.37(4.17,6.25) | 5.22(3.84,6.73) | 5.00(3.93,7.02) | 5.51(4.20,6.83) | 1.16 | 0.763 |
| cortisol | 333.40 (257.00, 487.21) | 315.95 (201.74, 436.53) | 262.53 (171.83, 355.43) | 244.54 (166.17, 367.11) | 13.32 | 0.004* |
| aldosterone | 9.47(6.27,13.12) | 8.63(6.18,11.68) | 6.88(5.62,10.08) | 7.15(4.98,9.70) | 18.00 | 0* |
| β-Hydroxybutyric acid | 15.92(12.66,32.81) | 17.13(11.65,28.88) | 16.47(12.59,25.78) | 20.88(12.42,47.13) | 1.90 | 0.594 |
| urine glucose | 14.20 (0.70, 99.20) | 29.40 (1.31, 161.94) | 51.50 (2.10, 183.64) | 21.16 (2.39, 125.85) | 2.12 | 0.548 |
| NAG | 16.00 (8.00, 28.00) | 16.00 (7.58, 31.00) | 16.00 (6.46, 26.00) | 11.66 (8.13, 30.50) | 0.34 | 0.952 |
| total protein | 7.10 (4.40, 13.30) | 8.10 (5.70, 16.45) | 10.80 (5.20, 20.10) | 16.45 (7.48, 59.85) | 16.39 | 0.001* |
| retinol-binding protein | 3.33 (1.55, 6.08) | 5.77 (0.93, 10.45) | 4.02 (0.47, 9.61) | 3.94 (0.26, 12.42) | 2.08 | 0.556 |
| Immunoglobulin G | 7.12 (3.56, 16.11) | 9.42 (5.21, 19.54) | 8.31 (5.18, 18.96) | 10.40 (5.96, 36.77) | 4.43 | 0.219 |
| Kappa Light Chain | 2.48 (1.26, 9.23) | 4.01 (1.82, 9.40) | 4.51 (1.74, 13.04) | 4.88 (2.64, 16.50) | 5.06 | 0.167 |
| Lambda Light Chain | 0.48 (0.24, 2.24) | 1.29 (0.53, 3.97) | 2.12 (0.64, 6.65) | 3.84 (0.91, 11.16) | 16.31 | 0.001* |
| Transferrin TRF | 9.38 (2.69, 17.38) | 13.65 (4.21, 20.74) | 9.59 (3.14, 21.30) | 12.18 (4.42, 20.58) | 2.06 | 0.56 |
| α1-microglobulin | 0.25 (0.02, 10.27) | 0.33 (0.07, 4.13) | 0.34 (0.07, 7.22) | 1.41 (0.05, 7.62) | 1.5 | 0.683 |
| β2-microglobulin U | 0.15 (0.10, 0.31) | 0.29 (0.14, 0.67) | 0.20 (0.09, 0.60) | 0.17 (0.04, 0.38) | 13.21 | 0.004* |

Note: * indicates a statistically significant difference at P < 0.05.

**6.Sex-Stratified Spearman Correlation Analysis Between Urinary Stress Hormones and Renal Function**

In sex-stratified analyses, urinary stress hormones demonstrated consistent associations with renal function, though the strength of correlations varied by sex. In males, NE and 17-KS showed significant negative associations with renal injury markers and positive associations with renal function. In females, NE exhibited even stronger negative associations with renal injury markers and positive associations with renal function, while 17-OHCS, COR, and ALD also showed significant sex-specific relationships.see**Table S7 and S8.**

**Table S7. Spearman Correlation Analysis Between Urinary Stress Hormones and Renal Function in Male Participants**

|  | UACR | UAMLA | SCr | BUN | eGFR |
| --- | --- | --- | --- | --- | --- |
| NE | -0.290** | -0.349** | -0.433** | -0.259** | 0.413** |
| E | 0.02 | -0.145** | -0.021 | 0.072 | -0.147** |
| HVA | -0.009 | -0.132** | -0.064 | -0.01 | -0.116* |
| VMA | -0.093 | -0.231** | -0.02 | -0.007 | -0.183** |
| 17-KS | -0.247** | -0.262** | -0.191** | -0.059 | 0.245** |
| 17-OHCS | 0.091 | 0.026 | -0.123* | -0.197** | 0.147** |
| COR | -0.132** | -0.177** | -0.217** | -0.098 | 0.117* |
| ALD | -0.122* | -0.213** | -0.171** | -0.069 | 0.079 |

Note：Significance levels: **p < 0.01,* p < 0.05

**Table S8. Spearman Correlation Analysis Between Urinary Stress Hormones and Renal Function in Female Participants**

|  | UACR | UAMLA | SCr | BUN | eGFR |
| --- | --- | --- | --- | --- | --- |
| NE | -0.124* | -0.217** | -0.475** | -0.304** | 0.391** |
| E | 0.024 | -0.169** | -0.179** | -0.029 | 0.009 |
| HVA | 0.068 | -0.074 | -0.223** | -0.035 | 0.097 |
| VMA | 0.007 | -0.091 | -0.084 | 0.047 | -0.044 |
| 17-KS | -0.067 | -0.167** | -0.246** | -0.189** | 0.202** |
| 17-OHCS | 0.016 | -0.062 | -0.336** | -0.195** | 0.345** |
| COR | -0.088 | -0.174** | -0.346** | -0.234** | 0.285** |
| ALD | -0.061 | -0.193** | -0.367** | -0.236** | 0.298** |

Note：Significance levels: **p < 0.01,* p < 0.05

1. **Sex-Stratified Univariate Logistic Regression Analysis**

In the sex-stratified analyses, the results of univariate logistic regression are provided in the **Supplementary Tables (Table S9–S10).** In males, traditional risk factors such as SBP, DBP, BMI, SCr, TG, and WBC were significantly associated with CKD with diabetes, alongside several tubular injury markers (TP, RBP, IgG, κ-LC, λ-LC, TRF, α1-MG, β2-MG). Notably, NE and 17-KS showed protective associations , whereas 17-OHCS emerged as a risk factor. In females, significant predictors included SBP, DBP, BUN, SCr, TG, and WBC, as well as tubular injury markers (TP, RBP, IgG, κ-LC, λ-LC, TRF, α1-MG). Unlike in males, stress hormones such as NE, 17-KS, 17-OHCS, and ALD were not significantly associated with CKD with diabetes risk in females.

**Table S9. Univariate Logistic Regression Analysis of Risk Factors Associated with CKD with diabetes in Male Participants**

| Exposure | B | SE | Wald | P | OR | LCL | UCL |
| --- | --- | --- | --- | --- | --- | --- | --- |
| DD | 0.036 | 0.017 | 4.623 | 0.032* | 1.037 | 1.003 | 1.072 |
| SBP | 0.039 | 0.008 | 22.323 | 0* | 1.039 | 1.023 | 1.056 |
| DBP | 0.032 | 0.011 | 8.067 | 0.005* | 1.033 | 1.01 | 1.056 |
| BMI | 0.098 | 0.036 | 7.279 | 0.007* | 1.103 | 1.027 | 1.184 |
| BUN | 0.097 | 0.051 | 3.617 | 0.057 | 1.102 | 0.997 | 1.218 |
| SCr | 0.019 | 0.004 | 21.948 | 0* | 1.019 | 1.011 | 1.027 |
| UA | 0.002 | 0.001 | 4.325 | 0.038* | 1.002 | 1 | 1.004 |
| Cys-C | -0.017 | 0.031 | 0.288 | 0.591 | 0.983 | 0.925 | 1.045 |
| Alb | -0.052 | 0.018 | 8.273 | 0.004* | 0.95 | 0.917 | 0.984 |
| DBil | -0.079 | 0.055 | 2.084 | 0.149 | 0.924 | 0.83 | 1.029 |
| TG | 0.151 | 0.052 | 8.328 | 0.004* | 1.163 | 1.05 | 1.288 |
| WBC | 0.092 | 0.045 | 4.239 | 0.04* | 1.096 | 1.004 | 1.196 |
| PLT | 0.005 | 0.002 | 8.128 | 0.004* | 1.005 | 1.002 | 1.009 |
| NE | -0.004 | 0.002 | 6.644 | 0.01* | 0.996 | 0.993 | 0.999 |
| E | 0 | 0.002 | 0.041 | 0.84 | 1 | 0.996 | 1.003 |
| VMA | 0.038 | 0.041 | 0.846 | 0.358 | 1.038 | 0.958 | 1.125 |
| HVA | -0.039 | 0.039 | 1.003 | 0.317 | 0.962 | 0.89 | 1.038 |
| 17-KS | -0.062 | 0.03 | 4.25 | 0.039* | 0.94 | 0.887 | 0.997 |
| 17-OHCS | 0.078 | 0.034 | 5.39 | 0.02* | 1.081 | 1.012 | 1.155 |
| COR | 0 | 0 | 1.001 | 0.317 | 1 | 1 | 1.001 |
| ALD | -0.004 | 0.02 | 0.042 | 0.839 | 0.996 | 0.959 | 1.035 |
| β-HB | 0 | 0 | 0.948 | 0.33 | 1 | 1 | 1.001 |
| UGLU | 0 | 0.001 | 0.125 | 0.724 | 1 | 0.999 | 1.002 |
| NAG | 0.004 | 0.005 | 0.794 | 0.373 | 1.004 | 0.995 | 1.013 |
| TP | 0.191 | 0.023 | 67.615 | 0* | 1.21 | 1.156 | 1.267 |
| RBP | 0.026 | 0.008 | 10.606 | 0.001* | 1.026 | 1.01 | 1.042 |
| IgG | 0.096 | 0.013 | 58.105 | 0* | 1.101 | 1.074 | 1.128 |
| κ-LC | 0.087 | 0.013 | 42.25 | 0* | 1.091 | 1.063 | 1.12 |
| λ-LC | 0.215 | 0.031 | 47.454 | 0* | 1.24 | 1.167 | 1.319 |
| TRF | 0.023 | 0.006 | 16.499 | 0* | 1.023 | 1.012 | 1.034 |
| α1-MG | 0.041 | 0.01 | 18.095 | 0* | 1.042 | 1.022 | 1.062 |
| β2-MG | 0.292 | 0.077 | 14.513 | 0* | 1.34 | 1.153 | 1.557 |

Note: * indicates a statistically significant difference at P < 0.05.

**Table S10. Univariate Logistic Regression Analysis of Risk Factors Associated with CKD with diabetes in Female Participants**

| Exposure | B | SE | Wald | P | OR | LCL | UCL |
| --- | --- | --- | --- | --- | --- | --- | --- |
| DD | 0.058 | 0.02 | 8.033 | 0.005* | 1.06 | 1.018 | 1.103 |
| SBP | 0.033 | 0.01 | 10.661 | 0.001* | 1.033 | 1.013 | 1.054 |
| DBP | 0.03 | 0.014 | 4.461 | 0.035* | 1.03 | 1.002 | 1.059 |
| BMI | 0.067 | 0.035 | 3.719 | 0.054 | 1.069 | 0.999 | 1.144 |
| BUN | 0.236 | 0.068 | 12.162 | 0* | 1.266 | 1.109 | 1.446 |
| SCr | 0.023 | 0.006 | 17.245 | 0* | 1.024 | 1.012 | 1.035 |
| UA | 0.002 | 0.001 | 2.839 | 0.092 | 1.002 | 1 | 1.004 |
| Cys-C | -0.032 | 0.036 | 0.818 | 0.366 | 0.968 | 0.902 | 1.039 |
| Alb | -0.035 | 0.023 | 2.331 | 0.127 | 0.965 | 0.923 | 1.01 |
| DBil | -0.126 | 0.077 | 2.69 | 0.101 | 0.882 | 0.758 | 1.025 |
| TG | 0.389 | 0.12 | 10.564 | 0.001* | 1.475 | 1.167 | 1.864 |
| WBC | 0.244 | 0.073 | 11.235 | 0.001* | 1.276 | 1.107 | 1.472 |
| PLT | 0 | 0.002 | 0.055 | 0.815 | 1 | 0.996 | 1.005 |
| NE | 0 | 0.001 | 0.121 | 0.728 | 1 | 0.998 | 1.002 |
| E | 0 | 0 | 0.268 | 0.605 | 1 | 0.999 | 1.001 |
| VMA | 0.043 | 0.029 | 2.199 | 0.138 | 1.044 | 0.986 | 1.105 |
| HVA | 0.018 | 0.02 | 0.839 | 0.36 | 1.018 | 0.98 | 1.058 |
| 17-KS | -0.047 | 0.036 | 1.72 | 0.19 | 0.954 | 0.889 | 1.024 |
| 17-OHCS | 0.032 | 0.038 | 0.727 | 0.394 | 1.033 | 0.959 | 1.113 |
| COR | -0.001 | 0.001 | 1.317 | 0.251 | 0.999 | 0.998 | 1 |
| ALD | 0.002 | 0.013 | 0.028 | 0.866 | 1.002 | 0.976 | 1.029 |
| β-HB | 0 | 0 | 0.282 | 0.595 | 1 | 1 | 1.001 |
| UGLU | 0.001 | 0.001 | 1.929 | 0.165 | 1.001 | 0.999 | 1.003 |
| NAG | 0.004 | 0.008 | 0.214 | 0.644 | 1.004 | 0.988 | 1.019 |
| TP | 0.114 | 0.018 | 42.066 | 0* | 1.121 | 1.083 | 1.16 |
| RBP | 0.034 | 0.013 | 6.696 | 0.01* | 1.034 | 1.008 | 1.061 |
| IgG | 0.116 | 0.017 | 45.58 | 0* | 1.123 | 1.086 | 1.161 |
| κ-LC | 0.139 | 0.024 | 34.578 | 0* | 1.149 | 1.097 | 1.204 |
| λ-LC | 0.399 | 0.059 | 46.365 | 0* | 1.49 | 1.329 | 1.672 |
| TRF | 0.036 | 0.01 | 13.581 | 0* | 1.037 | 1.017 | 1.057 |
| α1-MG | 0.102 | 0.022 | 21.465 | 0* | 1.108 | 1.061 | 1.157 |
| β2-MG | 0.022 | 0.031 | 0.52 | 0.471 | 1.022 | 0.963 | 1.086 |

Note: * indicates a statistically significant difference at P < 0.05.

**8.Sex-stratified Multivariate Logistic Regression Analyses**

In male participants (Table S11), independent predictors of CKD with diabetes included elevated SCr and DBP, decreased NE, and increased 17-OHCS.In female participants (Table S12), the predictors included elevated SCr and DBP, decreased NE, increased HVA, and decreased 17-KS.These results suggest that while both sexes share common predictors such as SCr, DBP, and NE, there are also sex-specific associations: 17-OHCS was uniquely predictive in males, whereas HVA and 17-KS were specific to females.

**Table S11. Multivariate Logistic Regression Analysis of Predictive Factors for CKD with diabetes in Male Participants**

| Exposure | B | SE | Wald | P | OR | LCL | UCL |
| --- | --- | --- | --- | --- | --- | --- | --- |
| SCr | 0.657 | 0.237 | 7.662 | 0.006* | 1.928 | 1.211 | 3.069 |
| Alb | -0.05 | 0.025 | 3.845 | 0.05 | 0.951 | 0.905 | 1 |
| DBP | 0.059 | 0.015 | 15.804 | 0* | 1.061 | 1.03 | 1.092 |
| NE | -0.574 | 0.208 | 7.593 | 0.006* | 0.563 | 0.374 | 0.847 |
| HVA | 0.081 | 0.178 | 0.21 | 0.647 | 1.085 | 0.766 | 1.537 |
| 17-KS | -0.073 | 0.167 | 0.188 | 0.665 | 0.93 | 0.67 | 1.291 |
| 17-OHCS | 0.635 | 0.194 | 10.722 | 0.001* | 1.887 | 1.29 | 2.76 |
| UGLU | 0.077 | 0.17 | 0.205 | 0.651 | 1.08 | 0.774 | 1.508 |

Note: * indicates a statistically significant difference at P < 0.05.

**Table S12. Multivariate Logistic Regression Analysis of Predictive Factors for CKD with diabetes in Female Participants**

| Exposure | B | SE | Wald | P | OR | LCL | UCL |
| --- | --- | --- | --- | --- | --- | --- | --- |
| SCr | 1.206 | 0.324 | 13.817 | 0* | 3.341 | 1.769 | 6.31 |
| Alb | 0.012 | 0.042 | 0.08 | 0.777 | 1.012 | 0.932 | 1.098 |
| DBP | 0.053 | 0.019 | 8.296 | 0.004* | 1.055 | 1.017 | 1.094 |
| NE | -0.538 | 0.263 | 4.177 | 0.041* | 0.584 | 0.349 | 0.978 |
| HVA | 0.62 | 0.23 | 7.244 | 0.007* | 1.859 | 1.184 | 2.92 |
| 17-KS | -0.408 | 0.186 | 4.818 | 0.028* | 0.665 | 0.462 | 0.957 |
| 17-OHCS | 0.351 | 0.24 | 2.142 | 0.143 | 1.42 | 0.888 | 2.271 |
| UGLU | 0.616 | 0.232 | 7.038 | 0.008* | 1.852 | 1.175 | 2.92 |

Note: * indicates a statistically significant difference at P < 0.05.

**Supplementary Discussion**

The heterogeneity of type 2 diabetes should also be considered when interpreting our findings. Previous studies have proposed distinct T2D subtypes, such as severe insulin-deficient diabetes (SIDD), severe insulin-resistant diabetes (SIRD), mild obesity-related diabetes (MOD), and mild age-related diabetes (MARD), which differ in their clinical characteristics and risk of complications(1). The relatively disperse distribution of urinary hormone levels in the DM group observed in our study may partly reflect this underlying heterogeneity. Since our primary aim was to investigate differences between DM and CKD with diabetes groups, we did not perform clustering analyses to stratify patients into these subtypes. Nevertheless, considering T2D heterogeneity may provide valuable insights in future studies and help to refine risk stratification for CKD with diabetes.

**References**

1. Ahlqvist E, Prasad RB, Groop L. Subtypes of Type 2 Diabetes Determined From Clinical Parameters. Diabetes. 2020;69(10):2086-93.
